# The importance of mass spectrometric dereplication in fungal secondary metabolite analysis

**DOI:** 10.3389/fmicb.2015.00071

**Published:** 2015-02-17

**Authors:** Kristian F. Nielsen, Thomas O. Larsen

**Affiliations:** Department of Systems Biology, Technical University of Denmark, Kongens LyngbyDenmark

**Keywords:** liquid chromatography, metabolomics, mass spectrometry, diode array detection, dereplication

## Abstract

Having entered the *Genomic Era*, it is now evident that the biosynthetic potential of filamentous fungi is much larger than was thought even a decade ago. Fungi harbor many cryptic gene clusters encoding for the biosynthesis of polyketides, non-ribosomal peptides, and terpenoids – which can all undergo extensive modifications by tailoring enzymes – thus potentially providing a large array of products from a single pathway. Elucidating the full chemical profile of a fungal species is a challenging exercise, even with elemental composition provided by high-resolution mass spectrometry (HRMS) used in combination with chemical databases (e.g., AntiBase) to dereplicate known compounds. This has led to a continuous effort to improve chromatographic separation in conjunction with improvement in HRMS detection. Major improvements have also occurred with 2D chromatography, ion-mobility, MS/MS and MS^3^, stable isotope labeling feeding experiments, classic UV/Vis, and especially automated data-mining and metabolomics software approaches as the sheer amount of data generated is now the major challenge. This review will focus on the development and implementation of dereplication strategies and will highlight the importance of each stage of the process from sample preparation to chromatographic separation and finally toward both manual and more targeted methods for automated dereplication of fungal natural products using state-of-the art MS instrumentation.

## INTRODUCTION

Filamentous fungi are prolific producers of secondary metabolites (SM) of importance to humankind. Useful fungal metabolites include drugs, food colorants, feed additives, industrial chemicals, and biofuels (Bills, 1995; Bode et al., 2002; Firn and Jones, 2003; Butler, 2004). Fungi are also known for their negative consequences as contaminants of food and feed due to the production of mycotoxins which can be cytotoxic, immunotoxic, estrogenic, or carcinogenic (Miller, 2008; Shephard, 2008). Fungi can also cause invasive human infections, especially in immuno-compromised individuals (Larsen et al., 2007).

Having entered the *Genomic Era* it is now clear that the biosynthetic and metabolic diversity potential of filamentous fungi, is still vast, due to the presence of many cryptic SM encoding gene clusters. This is particularly true for non-model organisms. The abundance of cryptic gene clusters has resulted in the use of many strategies to stimulate gene expression aimed toward the discovery of novel bioactive compounds and characterization of their biosynthetic pathways (Bode et al., 2002; Bergmann et al., 2007; Bok et al., 2009; Schroeckh et al., 2009; Sarkara et al., 2012; Sørensen et al., 2012; Droce et al., 2013; Sorensen and Sondergaard, 2014). Uncovering the full chemical potential of any micro-organism is a challenging exercise. Firstly, expression of metabolites related to a given gene cluster is highly regulated and may only be expressed under special condition, and furthermore each cluster may be responsible for more than 10 end products and a similar number of stable intermediates also present in detectable concentrations (Degenkolb et al., 2006; Chiang et al., 2010; Nielsen et al., 2011b; Ali et al., 2014; Holm et al., 2014; Petersen et al., 2015). In addition, crosstalk between biosynthetic pathways can result in compounds that are products of more than one gene cluster (Nielsen et al., 2011b; Tsunematsu et al., 2013). Overall this results in an extremely complex pool of diverse small organic molecules to identify by chemical analysis, especially when considering a single species of *Aspergillus* and *Fusarium* contains 50–80 gene clusters (Sanchez et al., 2012; Lysoe et al., 2014).

It is common for natural products to have identical elemental composition (up to 130 for some terpenes) making unambiguous identification very challenging. As such, without access to authentic standards, elemental composition alone – obtained by high resolution mass spectrometry (HRMS) –is not enough to unambiguously identify compounds (Nielsen et al., 2011a; El-Elimat et al., 2013). Consequently, fast identification of previously described compounds without reference standards – known as dereplication – is a very challenging task. As reference standards of most secondary metabolite standards are not available, fast dereplication is vital for progress in both drug discovery and pathway elucidation projects (Cordell and Shin, 1999; Dinan, 2005; Zhang, 2005; Bitzer et al., 2007; Feng and Siegel, 2007; Stadler et al., 2009; Zengler et al., 2009; Nielsen et al., 2011a). Dereplication is most often done by Ultra high performance liquid chromatography (UHPLC) coupled to diode array detection (DAD) and HRMS, in combination with database searching. Proper dereplication strategies ensures that time consuming and costly efforts with isolation and subsequent NMR based structure elucidation can be focused solely on novel compounds (Cordell and Shin, 1999) or that re-isolation of known compounds can be done in an efficient way based on functional groups and thus fewer steps (Månsson et al., 2010). This paper will highlight important issues and recent approaches toward fast and reliable dereplication of fungal NPs primarily based on UHPLC-DAD-HRMS techniques.

## THE IMPORTANCE OF SAMPLE PREPARATION

The chemical diversity which is found both between and within the many classes of secondary metabolites (e.g., polyketides (PKs), non-ribosomal peptides (NRPs) and terpenoids), makes it impossible to quantitatively extract all secondary metabolites from a given fungus using a single procedure. Consequently, sample preparation is an important aspect of secondary metabolite profiling and will, no matter the choice of method, lead to bias toward certain types of compounds. To avoid extracting too many polar media components, primary metabolites and sugars an organic extraction is needed using one or more water-immiscible solvents such as ethyl acetate (EtOAc; Nielsen et al., 2011a; Stadler et al., 2014), dichloromethane (DCM; Abdel-Mawgoud et al., 2008), or 1-butanol (Yokota et al., 2012). The latter is efficient for the extraction of lipopeptides but has a high boiling point (118^∘^C), thus requiring both N_2_ and heating for evaporation. The pH is vital for an organic extraction, as ionizable molecules will be extracted into the organic phase to a much higher degree in their neutral form than when in a charged state. As almost 50% of all described fungal NPs (Månsson et al., 2010) contains an acidic moiety, a low pH extraction is necessary in most cases but can be supplemented with a neutral extraction (e.g., for stability reasons). Solvents such as ethers (highly flammable and able to form explosive peroxides) as well the carcinogenic and environmental damaging chloroform (CHCl_3_) and carbon tetrachloride (CCl_4_) are being phased out. Methanol and ethanol are also efficacious but, due to their high polarity, also results in the extraction of large quantities of salts and polar interfering substances which can quickly clog analytical HPLC columns. On the other hand, methanol/ethanol extracts can also contain highly non-polar waxes, sterols and triglycerides, however, these can usually be flushed out of the column at 80^∘^C with a mixture of acetonitrile-isopropanol for 1 h. We have experienced that injection of 1 μl of crude methanol extracts from marine media clogged an LC-MS electrospray source after analyzing only four extracts.

For extraction with EtOAc an essential process is the centrifugation of the 2-phase system as hard as possible then leaving behind the common interfacial agglomeration containing cells. Drying EtOAc extracts with anhydrous Na_2_SO_4_ can also result in cleaner extracts as EtOAc can contain up to 8% water. A major pitfall in sample preparation is the possibility of unwanted chemical reactions taking place. For example, alcohols can form esters with carboxylic acids and lactones (e.g., in homoserine lactones, rubratoxins, statins) under acidic conditions and can also catalyze various intramolecular changes (Rundberget et al., 2004). Acid catalyzed reactions can be especially problematic when evaporating organic extracts, since the lower volatility of acids such as formic and acetic acids will result in upconcentration when using volatile solvents like EtOAc and DCM, especially in the presence of residual water. Examples we have observed include; up to 50% loss of patulin during evaporation from EtOAc (Boonzaaijer et al., 2005); loss of trichothecenes prior to derivatization for GC-MS analysis (Langseth and Rundberget, 1998); up to 80% loss of fumonisins due to binding to the silanol groups of non-derivatized glass with up to 80% lost (Nielsen et al., 2015). We have used a fast extraction procedure to minimize the risk of studying artifact peaks, this procedure involves extraction into acetonitrile-water (1:1; in a ultrasonication bath or a beadbeater for circa 10 min), followed by centrifugation and subsequent transfer of the centrifugate directly to an auto-sampler compatible vial for analysis (Mogensen et al., 2011).

In some cases samples need to be purified on small solid phase extraction (SPE) columns to remove chromatography impairing lipids and phospholipids (Degenkolb et al., 2006; Pucci et al., 2009) or abundant but common secondary metabolites (e.g., as is *Stachybotrys*; Hinkley and Jarvis, 2000; Andersen et al., 2002). But SPE can also be used to simplify very complex extracts into several fractions. For example, ion exchange SPE can be used to separate an extract into acidic, neutral and basic analytes (Månsson et al., 2010), which may resolve new peaks and simplify the mass spectra. For an orthogonal separation to the common reversed phase analytical separation, we prefer diol or amino-propyl normal phase SPE (Bladt et al., 2013; Petersen et al., 2015) as we have found them to better separate compound classes compared to, e.g., pure silica.

## ANALYTICAL SEPARATION

When grown on rich growth media fungi often produce in excess of 100 SMs (Stadler et al., 2007, 2014; Bertrand et al., 2013; Kildgaard et al., 2014; Klitgaard et al., 2014; Wolfender et al., 2015). With such complex mixtures it is practically impossible to acquire enough separation power to provide complete resolution of all individual metabolites and media components and allow for subsequent spectroscopic analysis. On balance, by far the best choice for separation is reverse phase (RP) chromatography, since it’s polarity is well suited to most SMs, especially with the emergence of more polar phases such as pentafluoro phenyl, biphenyl, phenyl (Kildgaard et al., 2014; Wolfender et al., 2015) and also columns with various embedded groups (Euerby and Petersson, 2005). Many vendors have introduced improved the solid core particles of their stationary phase, ensuring no diffusion through the particle center (e.g., Poroshell, Kinetex, Ascentis, and Cortecs). Furthermore, hybrid chemistry particles (e.g., Waters BEH) provide sharper peaks as well as reduced tailing due to secondary interactions at a wide pH range. Performing LC at low pH is statistically preferably as 50% of all secondary metabolites contain an acidic moiety, while ca. 10% have a basic moiety (Månsson et al., 2010). Since sharper peaks are obtained from the non-charged state of SMs, low pH is preferable in RP chromatography. Undoubtedly the most prevalent buffers/acidifiers used are formic and acetic acid (Varga et al., 2013). Although triflouroacetic acid (TFA) is commonly utilized to lower the pH, this has the consequence of significantly suppressing negative ionization MS detection. We have chosen formic acid as, in addition to its lower pK_a_, it suppresses microbial growth in solvent reservoirs to a greater extent than acetic acid. We have found that, in general, formic acid appears to give sharper peaks at lower concentrations than acetic acid, and thus lower suppression of the UV signal. Although the use formic acid to adjust the pH is generally satisfactory, for some compounds it results in poor ionization, for example type B trichothecenes (Sulyok et al., 2006). Formic acid may also not be optimal if the majority of analysis is performed in negative mode – in which case acetic acid performs better – or if the analysis is performed under non-acidic conditions (Sulyok et al., 2006; Zhang et al., 2012).

Comprehensive 2D LC (Dugo et al., 2009; Chen et al., 2012) have recently been introduced by several of the UHPLC manufactures and introduced some interesting possibilities for orthogonal separation through the use of a long high-polarity HPLC column (20–30 min run) in the first dimension and a short highly retentive small particle C_18_ in the second dimension (20–30 s runs).

Whereas RP chromatography is usually a suitable choice for the separation of the wide range of middle-polarity secondary metabolites mainly produced by fungi, smaller very polar compounds are usually poorly retained. As a result of the lack of retention this chemical window has been exploited to a much lesser degree due to difficulties with analysis. To better investigate compounds in this high polar and/or ionic window (compounds with LogD < -3) techniques such as HILIC (Sørensen et al., 2007; Creek et al., 2011), high-pH ion-chromatography (IC; van Dam et al., 2002; Kvitvang et al., 2014) or ion-pair chromatography (Lebrun et al., 1989; Werner, 1993; Magdenoska et al., 2013) are needed to sufficiently retain the analytes. Common to all these techniques is that the window of separation is much narrower than for the extremely versatile RP, thus requiring more optimization. A major benefit of these techniques can be found in that many NP chemists only examine organic extractable compounds thereby overlooking many interesting high polar compounds (Cannall, 1998; Dufresne, 1998; Shimizu, 1998; Månsson et al., 2010). Other alternative choices for retention of polar compounds include anion or cation mixed mode columns (Apfelthaler et al., 2008). Examples such as the Dionex Trinety or the PrimeSep columns can retain anionic and/or cationic analytes strongly while also performing conventional RP chromatography (Lammerhofer et al., 2008).

Though LC is the dominant technique for the separation of compounds from complex mixtures there are several alternatives. For volatile compounds such as terpenes or compounds (e.g., acids) that can be derivatized to heat stable analytes GC-MS can be an efficient choice. GC-MS can be beneficial both due to the superior separation (10-100 higher peak capacity) but also due to the reproducible EI^+^ ionization that can be employed. Furthermore, technological improvements such as comprehensive GC × GC are becoming more affordable and dedicated TOFMS detectors for GC are being developed delivering accurate mass (<5 ppm accuracy) as well as GC-APCI interfaces for molecular mass identification.

Most recently, significant technological advances have occurred in the previously neglected field of supercritical fluid chromatography (SFC). Improvements have been made to SFC with better columns and much enhanced reproducibility by both Waters and Agilent. Together with novel column systems, many for enantiomeric separation, we foresee that this technique will become very valuable for analysis of SMs from the mid-polar region to the extremely non-polar (e.g., waxes and sterol esters). The technology can now be used with UV detection and is especially well suited for APCI.

## DIODE ARRAY DETECTION

**FIGURE 1:**
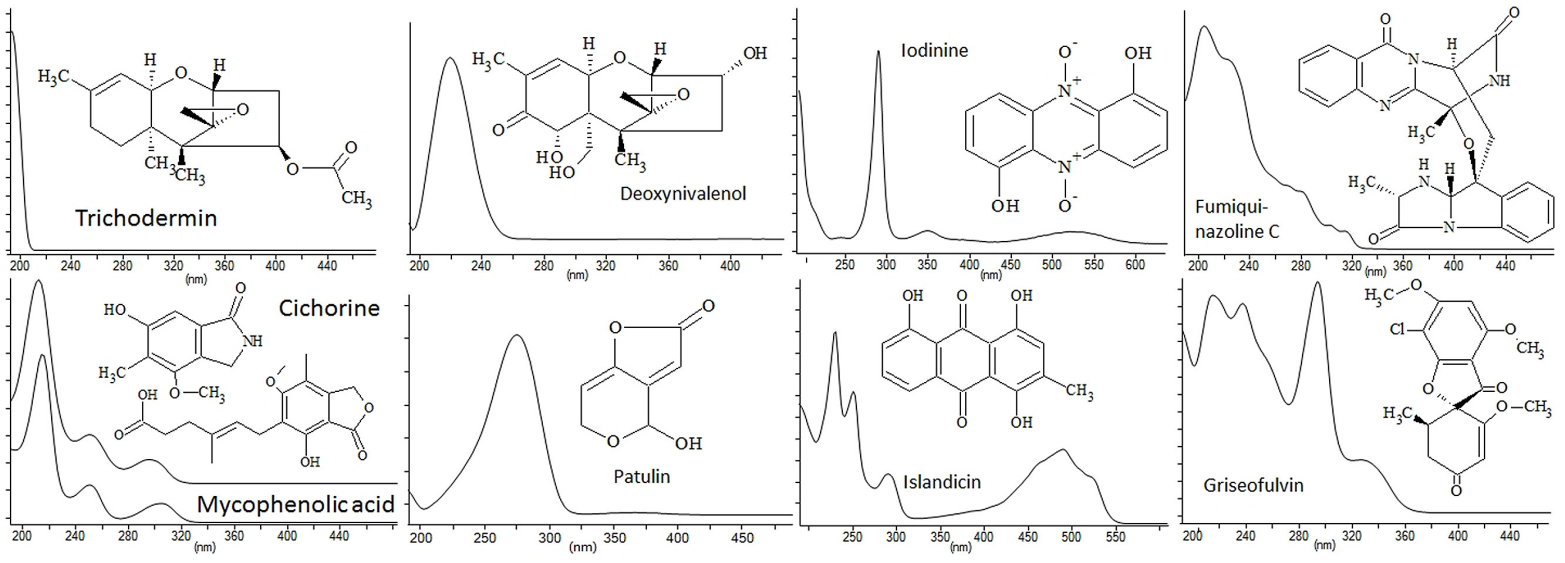
**UV/Vis spectra (pH 3) and structures of some common fungal natural products illustration the diversity in absorption range depending on the number of conjugated double bonds present in the molecules**.

Ultra high performance liquid chromatography MS is often combined with the relative inexpensive DAD as UV/Vis spectra provide information on chromophores which can be used for database searching (Hansen et al., 2005; Larsen et al., 2005; Bitzer et al., 2007; de la Cruz et al., 2012). More often DAD is used as second criteria after a MS based search, as neither AntiBase nor Dictionary of Natural Products provides a direct UV/Vis search from UV/Vis max data nor do they provide whole spectra. Many secondary metabolites, such as non-reduced PKs and NRPs contain conjugated chromophore systems. As such many metabolites have distinct UV-spectra (**Figure 1**) that are, in some cases, indicative of the basic skeleton of the given compound class, for example, quinazolines and γ-naphtopyrones produced by many *Aspergilli* and *Penicillia* (Larsen et al., 1999; Nielsen et al., 2009). Consequently, UV/Vis spectral chromophores can provide a means to differentiate compounds with the same elemental composition and can be highly valuable in dereplication for exclusion or confirmation of candidates during a database search. Though often useful, many compounds lack or contain few conjugated double bonds and only giving rise to one absorption band. Examples of compounds without diagnostic UV/Vis spectra include trichodermin, patulin, and deoxynivalenol (**Figure 1**). In cases such as these the UV/Vis spectrum is of little or no use. Other compounds, such as mycophenolic acid, contain a highly diagnostic chromophore (**Figure 1**), with three absorption maxima. Despite this informative finger print, compounds like mycophenolic acid also require MS verification due to the existence of at least 55 known compounds containing the 5,7-dihydroxy-methylphtalide core of mycophenolic acid and four with the cichorine core, as revealed by a substructure search in AntiBase 2012.

Analysis of UV/Vis spectra can also be very powerful for biosynthetic pathway elucidation studies, since compounds which belong to the same pathway often contain the same chemical scaffold and thereby chromophore system. This phenomenon was recently demonstrated during elucidation of the yanuthone biosynthetic pathway (Holm et al., 2014). Furthermore, DAD can be very useful for detection and discovery of compounds of the same type across species and genera, especially in cases where a certain class of compound has shown great promise as a new potential drug lead. Hansen et al. (2005) have developed an algorithm X-hitting for automated comparison of full UV spectra from LC-DAD analysis against a UV-library of standards as well as spectra across samples. This allows for both the identification of known compounds as well as new compounds with UV spectra similar to known compounds (Hansen et al., 2005; Larsen et al., 2005). Altogether DAD is a cheap and complementary spectroscopic technique easily performed in combination with full scan MS. Interestingly a recent software made in the same R environment^1^ as the popular XC-MS package (*vide infra*) for LC-MS, is now also available for LC-DAD (Wehrens et al., 2014).

Other (non-MS) detectors which are often used in combination with LC are evaporative light scattering detectors (ELSD; Bitzer et al., 2007; Yang et al., 2014) and the Corona cad detectors. These detectors give more quantitative information about the amount of compounds eluting from the LC unit, in contrast to MS detectors which are highly biased.

## MASS SPECTROMETRY

**FIGURE 2:**
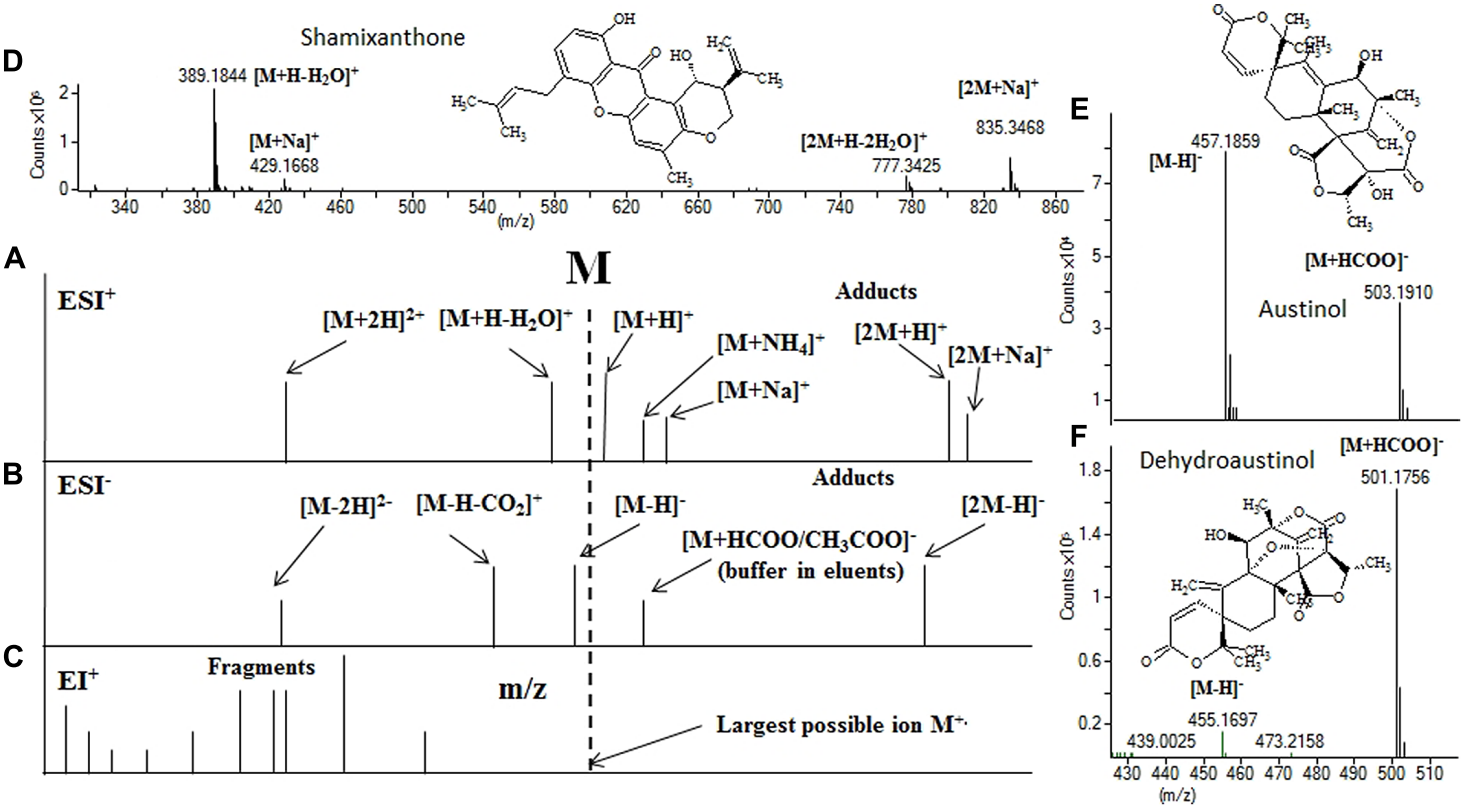
**Position of ions relative to the monoisotopic mass, of ESI^+^ (**A**), ESI^-^ (**B**) and EI^+^ (**C**) along with adduct ions, and 3 example ESI spectra.** In shamixanthone **(D)** the [M+H]^+^ ion is absent but the molecular mass can easily be assigned using the Δ40 (39.9925) between the [M+Na]^+^ and the [M+H-H_2_O]^+^. In **(E,F)** the big difference of formate adduct intensity on the austinols (same run, elutes 0.4 min in-between) is illustrated. For instrument settings see Kildgaard et al. (2014).

Today all MS instrumentations coupled to LC rely on atmospheric pressure ionization (API) techniques such as electrospray ionization (ESI), atmospheric pressure chemical ionization (APCI), or atmospheric pressure photo ionization (APPI). These are all soft ionization techniques, as such only limited fragmentation fungal SMs takes place at standard ionization conditions compared to what is seen when using hard ionization such as electron impact (EI) which is used for gas chromatography. Although limited fragmentation is usually observed, the default ion-source settings from many of the LC-MS manufactures, especially for ESI, cause quite extensive fragmentation to occur for very low molecular mass SMs (<300 Da) on most LC-MS systems (Nielsen et al., 2011a; Klitgaard et al., 2014; Rasmussen et al., 2014). Thus can the task of identifying the molecular mass in an API generated spectra become quite troublesome as a mix of fragment ions adducts and dimeric and double charged ions may be generated (**Figure 2**; Nielsen et al., 2011a; Kildgaard et al., 2014; Klitgaard et al., 2014). Consequently, the task of identifying the molecular mass in API generated spectra comes down to interpretation of the adduct patterns that can be seen for the different types of secondary metabolites (**Figure 2**).

Correct assessment of the molecular mass (M) is often not trivial as in-source fragmentation and adduct formation can lead to incorrect assignment of ions such as [M+Na]^+^, [M+NH_4_]^+^ or [M-H_2_O+H]^+^ as [M+H]^+^ (Nielsen et al., 2011a) or due to the low abundance of the [M+H]^+^ ion due to poor ionization under the selected conditions. A combination of both ESI^+^ and ESI^-^ ionization will assign the mass unambiguously, while just one polarity will only assign the mass unambiguously, when several adducts in a given mass spectrum all points toward the same molecular mass (Nielsen and Smedsgaard, 2003; Nielsen et al., 2011a). When the mass, or even better elemental composition, has been determined, this information can be searched against the literature to determine whether the compound is likely known or novel. *AntiBase* is currently the most suitable database for dereplication of fungal secondary metabolites as it only contains microbial metabolites. The 2014 version contains 43000 recorded compounds, including ∼18000 fungal and 21000 bacterial compounds. *Dictionary of Natural Products* additionally contains a large number of plant metabolites (some of which are produced by endophytic fungi and not the plant themselves), thus is this database a valuable addition to AntiBase. The databases Scifinder/CAS and RSC/Chemspider are highly biased toward synthetic compounds, and of less value. Furthermore hits in these databases cannot be sorted by organism type (Lang et al., 2008; Nielsen et al., 2011a). For Marine derived fungi MarineLit (Blunt et al., 2008) may be of interest and now available via RSC/Chemspider^2^.

In recent years data handling software has become the most crucial component of successful dereplication. Modern UHPLC-MS instruments can provide 50–200 data files per day with MS and tandem MS data, this can take many hours if not days per data file to manually handle, thus mandating more automated dereplication approaches. As with manual dereplication, the indisputable first step in automated dereplication is determination of the elemental composition of the individual compounds in the sample. For this accurate mass is vital. Depending on the mass range and the instrumental accuracy the elemental composition can often be determined unambiguously up to 300 Da at an accuracy of 5 ppm, while 1–2 ppm is required for compounds with masses up to 5–600 Da (Kind and Fiehn, 2007; Nielsen et al., 2011a). When accurate isotope ratio assessment is also reliable, it is possible to eliminate close lying elemental composition candidates with different numbers of carbon atoms (Bitzer et al., 2007; Lehner et al., 2011; Nielsen et al., 2011a; Bueschl et al., 2012; Sleno, 2012; Xian et al., 2012; Nagao et al., 2014).

Today the market is dominated by time of flight (TOF), Orbitrap, and Fourier transform ion cyclotron (FT-ICR) based mass spectrometers. TOF instruments deliver both superior isotopic ratios as well as an unmatched scan speed providing ample time for concurrent MS/MS experiments or the possibility of ultra-fast separation runs. A serious drawback of TOF based instruments is overloading of the detector leading to poor mass accuracy, which was very pronounced on the earlier TDC type detectors. Even for the newest 4 GHz ADC detectors overloading is still a problem and TOF related software needs to limit analysis to scans from the non-overloaded parts of the chromatographic peak (Kildgaard et al., 2014). Overall this results in poorer mass resolution than the two other common types of HRMS instruments. The FT-ICR technology provides an unsurpassed mass accuracy and resolution, for example an FT-ICR can easily resolve the A+2 ion of sulfur containing ions into the ^12^C_x_^13^C_2_^32^S and ^12^C_x+2_^34^S isotopomers. Unfortunately FT-ICR instruments suffer from high running costs for magnets, high acquisition price, as well as slow scan capability. The practical limitations of FT-ICR instruments have made Orbitrap type instruments much more popular as they do not require the expensive cooling of the superconducting magnets. In contrast to TOF based instruments Orbitraps do not suffer from the detector overload and needs less frequent mass calibration, though they deliver significantly slower scan speeds. The linear ion-trap (LIT)-orbitrap types can provide MS^n^ for fragmentation trees due to a dual detector providing both high and low resolution spectra, while Quadrupole (Q)-Exactive provides MS/MS as a QTOF and better quantitative performance than the Lit-Orbitrap, as well as the possibility of positive/negative switching.

Some of the earlier Orbitrap types used an ion-trap for fragmentation and helium as collision gas, which does not provide the same fragment ions as heavier gasses. Furthermore, ion-trapping usually has a limited m/z trapping window, a limitation that have been overcome by adding an additional collision cell on newer models (Perry et al., 2008). With respect to MS/MS spectra obtained from QqQ and QTOF instruments, we find the Q-Orbitrap to be superior. A class of lower cost instruments, which can perform MS^n^, are ion-trap (IT) spectrometers, albeit with low mass accuracy. This is very useful for peptide identification, and for other polymeric molecules made up of known units. In regard to dereplication, the common occurrence of chromatographic peaks indicating an unknown compound renders IT instruments inferior to accurate mass instruments. Triple quadrupole instrumentations have very poor full-scan sensitivity and are cost wise a poor choice for dereplication applications, but highly valuable for environmental analyses where sensitivity is the most important parameter (Sulyok et al., 2006; Sørensen et al., 2009).

For a number of years ion-mobility has been used in a range of configurations, for example, in the Waters Synapt instrument used for differentiating drift times of the fragment ions after MS/MS, however this has limited applications in SM analysis. Recently (May et al., 2014), a much higher resolution ion-mobility interface (positioned between the API source and a QTOF) has been disclosed and appears extremely promising to resolve co-eluting compounds which often have very different masses and cross sectional areas. Deconvolution of chromatographically co-eluting compounds can occur as the pseudo molecular ions and simple fragment ions (e.g., [M+H-H_2_O]^+^) have almost the same cross-section and thus same drift times. In contrast the co-eluting compounds are likely to have different cross sectional area and different drift times so can then be deconvoluted in the drift time dimension. This will provide a cleaner MS spectrum and more reliable interpretation of the molecular mass. Theoretically, a three dimensional deconvolution using both retention time and drift time can be used, but has to our knowledge not been developed.

## AUTOMATED TARGETED ANALYSIS

An alternate dereplication strategy is to search LC-MS data files for the masses (preferable accurate masses or elemental compositions) of a list of possible known compounds. This strategy, named aggressive dereplication, has been shown to work well for lists up to 3000 compounds (Kildgaard et al., 2014; Klitgaard et al., 2014), using the software from several manufactures. A major challenge is fragile compounds that easily fragment in the ion-source and thus appear as another elemental composition, leading to erroneous compound identification. The technology can be improved tremendously by introducing pseudo MS/MS in a second scan trace, so the instrument alternates between high and low fragmentation during a run (MS-All, MS-E, All-Ions; Ojanpera et al., 2006; Broecker et al., 2011; Guthals et al., 2012; El-Elimat et al., 2013) to obtain compound specific fragment ions known from our own standards, literature data, or from *in-silico* fragmentation (Hill and Mortishire-Smith, 2005; Kangas et al., 2012; Hufsky et al., 2014). The MS-All, MS-E, All-Ions strategy also has the advantage that the [M+Na]^+^ is often enhanced in the fragmentation trace and an accurate confirmation of [M+Na]^+^ to [M+H]^+^ or [M+H-H_2_O]^+^ cuts down on the number of false positives resulting from misidentification of in-source fragment ions (e.g., [M+H-H_2_O]^+^ being identified as [M+H]^+^ of another compound). Here we have observed that using an algorithm locking the pair like Bruker Target 1.3, Agilent Quant (*vide infra*), or ACD/Spectrus Processor provides 2–10 fold less false positives than Agilent Qual (Kildgaard et al., 2014) or Bruker Target 1.2 (Klitgaard et al., 2014) where one cannot require pairs of [M+Na]^+^ and [M+H]^+^ or [M+H-H_2_O]^+^.

## CONSTRUCTION OF COMPOUND DATABASE

An important part of targeted analysis is construction of the compound database to be used for searching. Kildgaard et al. (2014) and Klitgaard et al. (2014) used ACD Chemfolder (Advanced Chemistry Development, Toronto, ON, Canada) for construction of a database including: (i) a number of in-house reference standards; (ii) a selection of common fungal compounds from AntiBase; (iii) common impurity compounds known from blank samples or as common media components, and finally iv) a number of tentatively identified compounds. Compound-database handlers like Chemfolder or Chemfinder have the advantage of the ability to perform substructure searches, which is why these are preferred for a master database. For each compound, major adducts (known or predicted) were registered in the database [41]. If known and/or predicted fragment ions from an alternating broad band fragmentation (MS-E, All-Ions) are included the specificity is greatly increased.

The next step is to create a taxonomically relevant search list (if chemotaxonomic data from properly identified fungal identifications are available). If a species specific search list is not available data from the whole genus may be used depending on the number of compounds described. However, a balance must be found between the number of false positives from compounds with the same elemental composition and with compounds failing to be identified and requiring subsequent manual deconvolution.

Minor in-house programming (e.g., Excel) is required to transfer from a Chemfolder/Chemfinder database to a ready search list for the MS vendor software (Bruker Target analysis (Klitgaard et al., 2014), Agilent Masshunter-find by formula (Kildgaard et al., 2014), or Waters ChromaLynx etc. Different settings for retention time, mass accuracy of peaks, area cut-off, among others can be used to process data-files depending on the nature of the samples.

**FIGURE 3:**
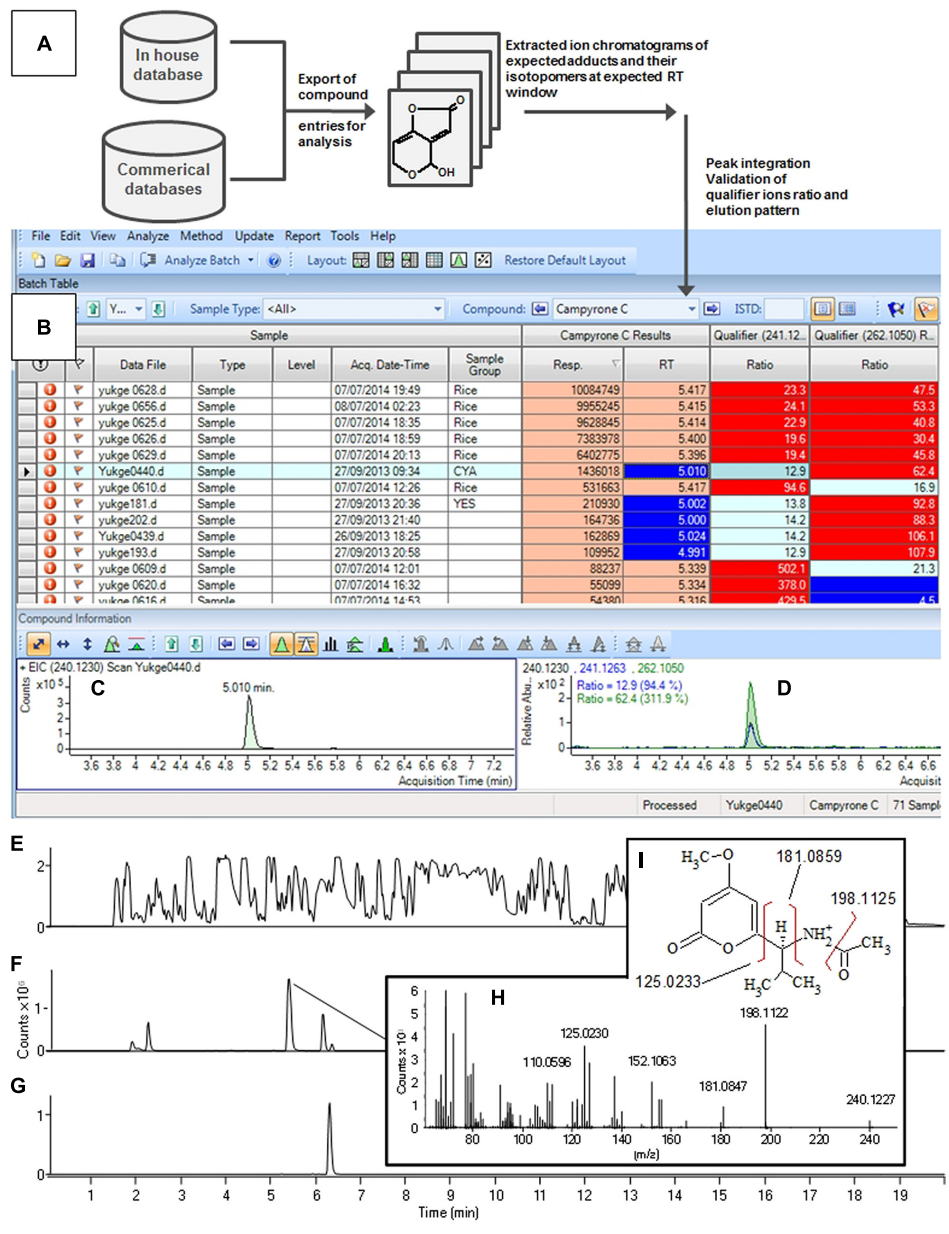
**Aggressive (A–C) batch processing of 150 samples (12 shown), here sorted by peak area (Response column) of mass of [M+H]^+^ of campyrone C (240.1230 ± 20 ppm) shown in (C).** While **(D)** shows the overlaid extracted ion chromatograms of the same as well as ^13^C_1_^12^C_12_ [M+H]^+^ isotopomer and the [M+Na]^+^ ion. **(F–I)** Manual verification of data file with highest peak intensity of campyrone C. **(E)** Shows the highly complex Base Peak chromatogram, while, the extracted ion chromatograms of [M+H]^+^ of campyrone C and A/B respectively. **(G)** Shows the MS/MS spectrum at 10 eV of the tentatively identified campyrone C with **(H)** Showing the structure of campyrone C along with **(I)** theoretical masses of 3 likely fragmentation of the molecule. For instrument settings see Kildgaard et al. (2014).

In some concurrent large screening studies of black aspergilli, we have used a multi-sample screening tool, Agilent MassHunter Quant (similar packages available from Waters and Thermo), developed for LC-QqQ targeted quantification of pesticides, for example. Despite being outside the original design the software can also handle high-resolution data. Thus a target list can be imported via an XML file and one can convert a Chemfolder/Chemfinder database (e.g., [M+H]^+^, [^13^C_1_M+H]^+^ [M+Na]+, [^13^C_1_M+Na]^+^, with RT window and fragment ions) to an XML file and import into MassHunter Quant (**Figures 3B–D**).

The advantage of the multi-sample screening approach is that whole batches of anywhere from 10 to 100s of samples including blanks, fungal strains grown on multiple media can be screened simultaneously. This not only gives fast processing, but a high chance of identifying a compound not previously detected while concurrently identifying the most prodigious producer of the batch. In many cases several peaks of interest are identified but with a proper selection of qualifier ion and comparisons between species and media and blanks usually only 2–3 peaks in the whole batch require further manual inspection. Here the sample with the most intense peak can be selected automatically as it is most likely to provide the best MS/HRMS and possible UV/Vis data. This is illustrated in **Figure 3** where a part of the batch of samples is shown, here screening for campyrone C, using [M+H]^+^ (240.1230 ± 20 ppm, **Figure 3C**). **Figure 3D** shows the qualifier chromatograms, including [M+Na]^+^, showing that the compound has the right elemental composition. The compound was then manually verified (**Figures 3E–H**) through conclusive MS/HRMS spectra and a correct elution order of the A or B isomers.

Altogether, the target analysis approach makes it possible to easily identify chromatographic peaks which are both likely to represent already known compounds and, even more importantly, also peaks that do not correspond to known compounds. Thus the target approach can quickly support prioritization in relation to which compounds might need to be produced in larger scale for semi-preparative isolation and possibly full structural characterization based on isolation and NMR spectroscopy. In our view this approach is very well suited to fungi that has already been studied significantly and when, for example, a new species related to a well characterized species is to be investigated.

## PEAK-FINDING

A more classical approach to investigation of a full scan UHPLC-HRMS file is to use a peak finding algorithm based on molecular features. This approach uses deconvolution of the time profiles and adds peaks together and possibly resolves the adduct pattern (Kuhl et al., 2012; Kildgaard et al., 2014). Most vendor software as well as the open source software XC-MS (Smith et al., 2006) with the Camera package (Kuhl et al., 2012) offers this feature although not all can search Chemfolder/Chemfinder Structure Data Format (SDF). However they can all search ChemSpider and similar public databases. This approach is probably best suited to extracts from species where the taxonomy is not well known and can be used in a true unbiased metabolomics workflow (Cox et al., 2014; Macintyre et al., 2014; Wolfender et al., 2015), and can be especially useful when combined with bioactivity or gene knock-outs.

A major obstacle for this method is that *all* samples to be compared need to be processed at the same time, as changes in media batches, impurities in solvents, filters, plastic and glassware (often strongly ionizing impurities) strongly influences data analysis. In addition to this there are problems associated with the likelihood of changes in secondary metabolism which are often seen for many organisms, even though great efforts are made to standardize cultivation conditions. Finally analytical separation, cleanliness of ion-sources, changes in LC-MS solvents and many other things also may cause changes in data. One can standardize data between batches but this demands an enormous amount of quality assurance work as it needs to include at least all the variable things mentioned above and is likely to still result in large uncertainties.

Thus, if using a metabolomics approach to find small peaks in extracts, a very strong experimental design, with 4–6 replicates per condition is needed, and ideally all samples need to be processed and analyzed in a single batch. If this strict control is not implemented the peak picking algorithm and unbiased data analysis will likely find the sample preparation day as the most significant parameter, especially when lowering the intensity threshold. In such cases 5,000–30,000 chemical features (Cox et al., 2014; Macintyre et al., 2014; Wolfender et al., 2015) may be detected and statistically some will always be correlated to a given hypothesis. Despite these shortcomings, for extracts with very high media background and very low signal intensity from the secondary metabolites this approach is the most effective. In our view this approach is highly time-consuming and if searching for a bioactive compound, an assay guided approach may be far less time consuming.

**FIGURE 4:**
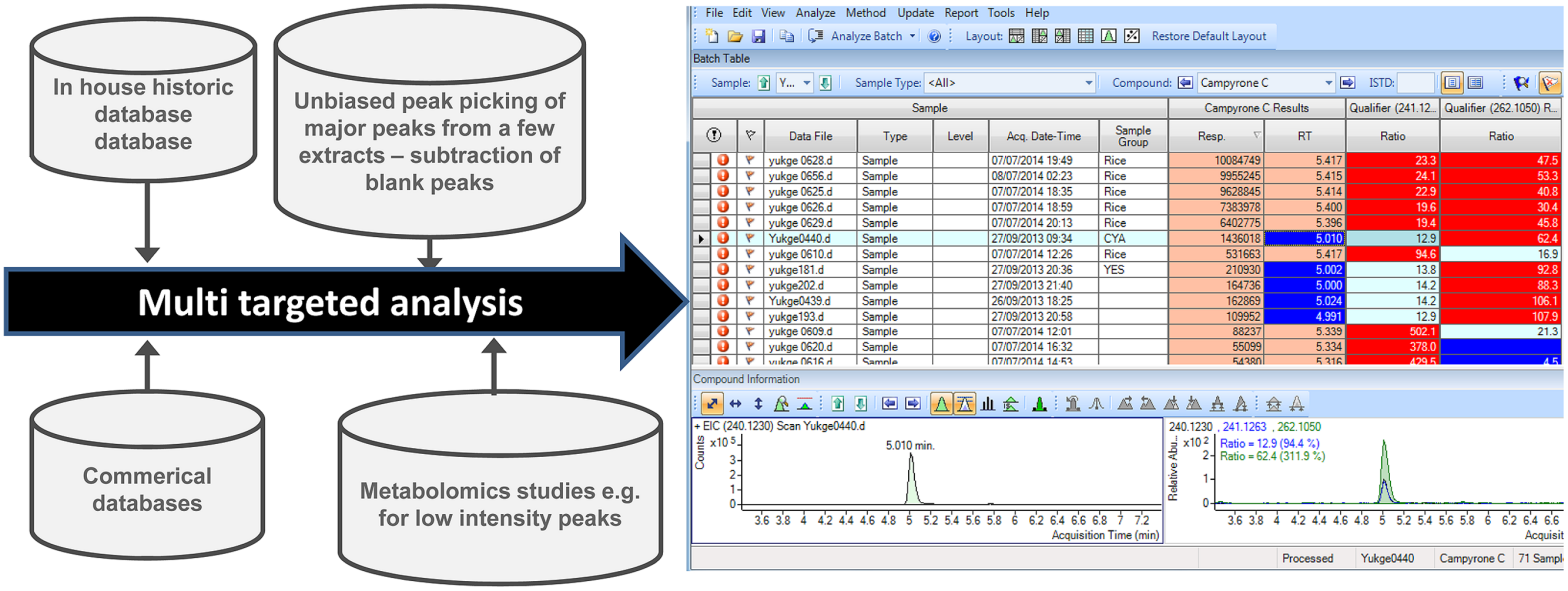
**Suggested workflow in fungal natural products research depending on the problem, number of strains, species and of peaks are minor or major, as well as if it is as well-studied or new organism, the different data-parts may differ significantly**.

The metabolomics workflow is often not easily implementable with the workflow in a natural product laboratory as different species and strains often need substantial optimization in terms of growth and analysis (**Figure 4**). However, for gene-to-product linking type of projects, it can be the only way to search for low intensity peaks in defined studies, as one cannot add samples from later studies.

## API MS/MS LIBRARIES

As mentioned above, UHPLC-HRMS is often not enough for a positive identification and a library based identification – as is possible for EI^+^ fragmentation – would be highly desirable. If matching LC-MS generated MS spectra to a library they must be reproducible to give reliable matches. This is not possible with full scan MS due to the differences in in-source fragmentation settings and adduct formation and spectra of the same compound can be very different even on the same instrument. However MS/MS spectra of the pseudomolecular ions, in general, produces the same fragment ions, although the fragmentation energy to provide the same spectrum may be very different and also will vary between the harder argon toward the softer nitrogen (Baumann et al., 2000; Fredenhagen et al., 2005; Lee et al., 2005; Pavlic et al., 2006; Oberacher et al., 2012; El-Elimat et al., 2013; Kildgaard et al., 2014).

Thus an additional scan type, MS/MS, can be used simultaneously with full-scan, when using a QTOF, IT-Orbi-Trap, Q-Orbi-Trap, as these can perform auto-MS/MS, or data dependent MS/MS. While TOF and Orbi-trap (Excactive) instruments cannot isolate an ion for MS/MS, they can perform broad band excitation (All-Ions or MS-E) fragmentation at some point in the ion-optics. A corollary is that fragmentation in an ion-trap often provides very different fragments than a collision cell as fragment ions that are outside the excitation window in the ion-trap are no longer accelerated and thus fragmented. To remedy this shortcoming some Orbi-Trap type spectrometers come with an additional hexa- or octapole collision cell.

Compared to forensic science and toxicology (Broecker et al., 2011) only few in-house MS/MS libraries with fungal metabolite spectroscopic data are available thus far (Kildgaard et al., 2014). The major reasons are firstly; the lack of requirement to publish MS/HRMS spectra and secondly; the lack of standardization of fragmentation energies between instrument manufactures. Although only containing relatively few microbial natural products, the two libraries that can be helpful, to identify lipids, medium polar primary metabolites, vitamins, and other coumpunds are Massbank (Horai et al., 2010) and Metlin (Smith et al., 2005; ∼10,000 compounds with spectra).

In addition it is unreliable to use *in silico* predictors for prediction of fragmentation of NPs, since they often represent highly condensed and complex ring structures, only leaving room for verification of some fragments from a structure in a spectrum (Hill and Mortishire-Smith, 2005; Hufsky et al., 2014). Despite of these challenges we anticipate that the use of MS/HRMS spectral libraries will become much more pronounced in the near future, especially since the different vendors can see a huge advantage in being able to supply their customers with dedicated spectral libraries.

### PRECURSOR SELECTION

The use of MS/MS on one of the pseudomolecular ions does indeed already provide very reproducible MS/MS spectra within one brand of instrument (Fredenhagen et al., 2005) as recently demonstrated in our laboratory (Fredenhagen et al., 2005; Kildgaard et al., 2014). Thus, good reproducibility can be achieved if the collision energy is calibrated and the nature of metal ion adducts are known, since [M+Na]^+^ and [M+K]^+^ fragment far less and with different mechanisms than [M+H]^+^, [M+NH_4_]^+^ (Oberacher et al., 2009, 2011; Wurtinger and Oberacher, 2012; Kildgaard et al., 2014). For robustness, many of the known adduct ions should be included as it increases the identification confidence especially between related compounds (Kildgaard et al., 2014).

### FRAGMENTATION

Importantly, different energies for MS/MS are needed as the stability of compounds varies significantly (Kildgaard et al., 2014). This can be acquired in two ways: (i) as several distinct energies (Smith et al., 2005), or ii) by ramping the collision energy, thus acquiring an average spectrum. For fungal metabolites we have found the spectral quality (Kildgaard et al., 2014) to be much higher when using distinct energies, especially when using accurate mass of the fragment ions. In such cases only 3–7 fragment ions are needed, as long as they are distributed equally over the mass range (neither in the very low range, with common fragments shared with many other compounds, nor close to the molecular ion where the losses may not differentiate from related compounds). Agilent Technologies and Metlin have chosen to acquire spectra at the three different fragmentation energies 10, 20, and 40 eV (Kildgaard et al., 2014), which we also found efficient for microbial metabolites, except for large peptides where 60 eV had to be included, and we suggest a possibility to change the window for these when going to larger masses where much more energy is generally needed.

### ALGORITHMS AND LIBRARY SCORING

**FIGURE 5:**
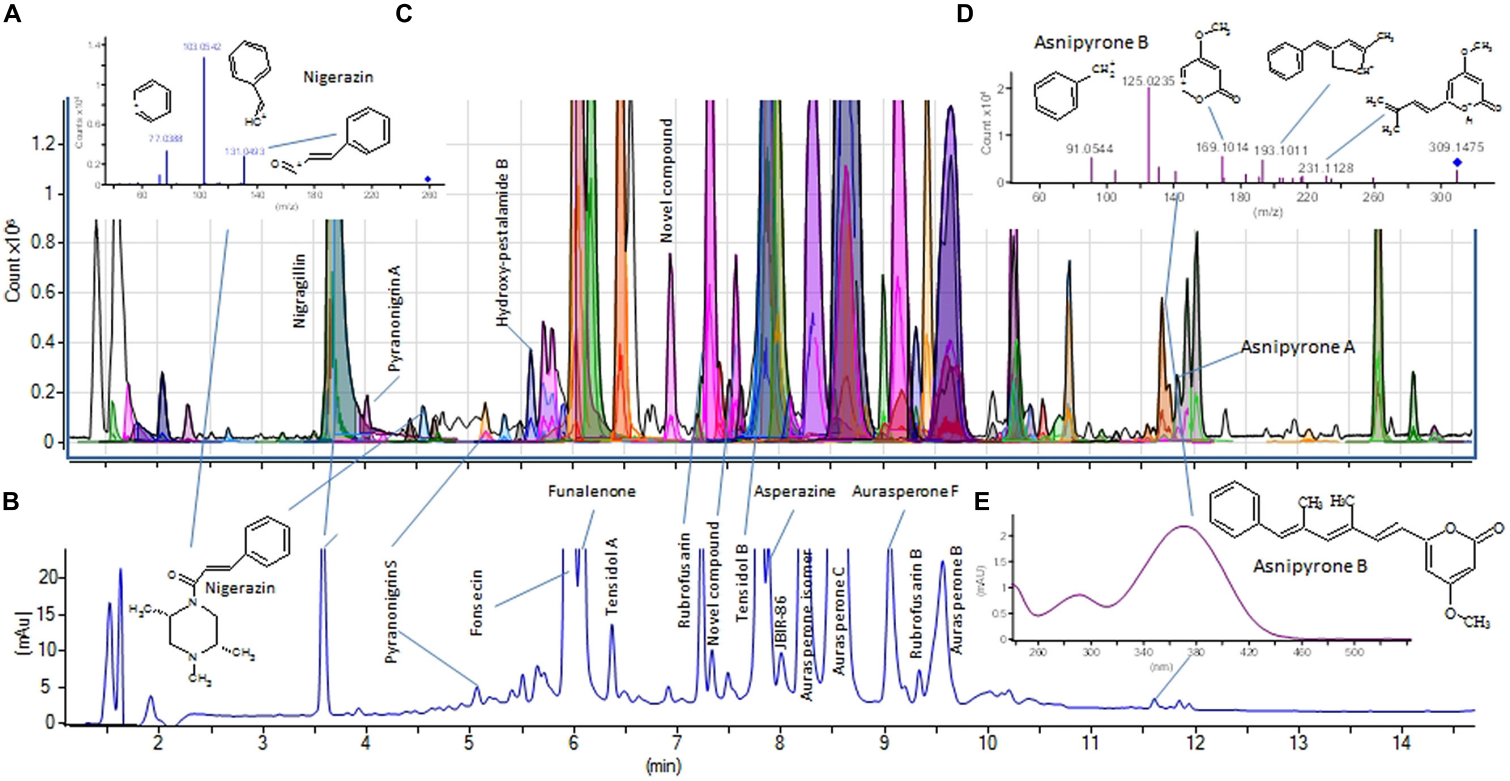
**UHPLC-DAD-HRMS-MS/HRMS analysis of an *Aspergillus tubingensis* extract. (A)** shows the BasePeak chromatogram. Peaks automatically assigned from Auto-MS/MS data searched in the MS/HRMS database are colored. **(B)** shows the UV/Vis chromatogram (200–640 nm), while **(C)** shows the MS/HRMS spectrum of nigerazin A (20 eV) or B used to tentatively ID this peak. **(D)** and **(E)** shows the MS/HRMS (20 eV) and UV/Vis spectra of asnipyrone B, respectively, with the UV/Vis spectrum matching literature data perfectly, and also eluting before asnipyrone A (having an extra methyl group). For instrument settings see Kildgaard et al. (2014).

Different algorithms have been used to search experimental MS/MS spectra contained in small in-house MS/MS libraries for tentative identification of fungal SMs. For example the NIST (National Institute of Standards and Technology) algorithm, developed for full scan EI^+^ spectra and the Mass Frontier software for MS^n^ spectra were compared by Fredenhagen et al. (2005) to search low resolution MS/MS data, with the latter found to be superior. Similarly El-Elimat et al. (2013) used ACD-IntelliXtract, which allows inclusion of accurate masses of the fragments, but does not use the parent ion data as search entry [64]. We use the Agilent search algorithm that is an integral part of the Agilent MassHunter software for fast and automated search in our in-house MS/HRMS library of more than 1300 compounds for unambiguous identification of especially fungal metabolites belonging species in the genera *Aspergillus*, *Penicillium*, and *Fusarium* (**Figure 5**).

The software allows background subtraction and merging of spectra over chromatographic peaks into a single spectrum prior to automatic searching against the library. Importantly, searching of MS/HRMS spectra against a given in-house library in MassHunter allows for both forward and reverse scoring using the parent mass for matching of peaks in the unknown spectrum against the library spectra or vice versa. Often both forward and reverse scoring are needed for correct identification as shown in our work for the identification of patulin (Kildgaard et al., 2014). Part of our library (277 microbial compounds) can be downloaded as PCDL format from the homepage of the Technical University of Denmark (Nielsen and Frisvad, 2014).

**FIGURE 6:**
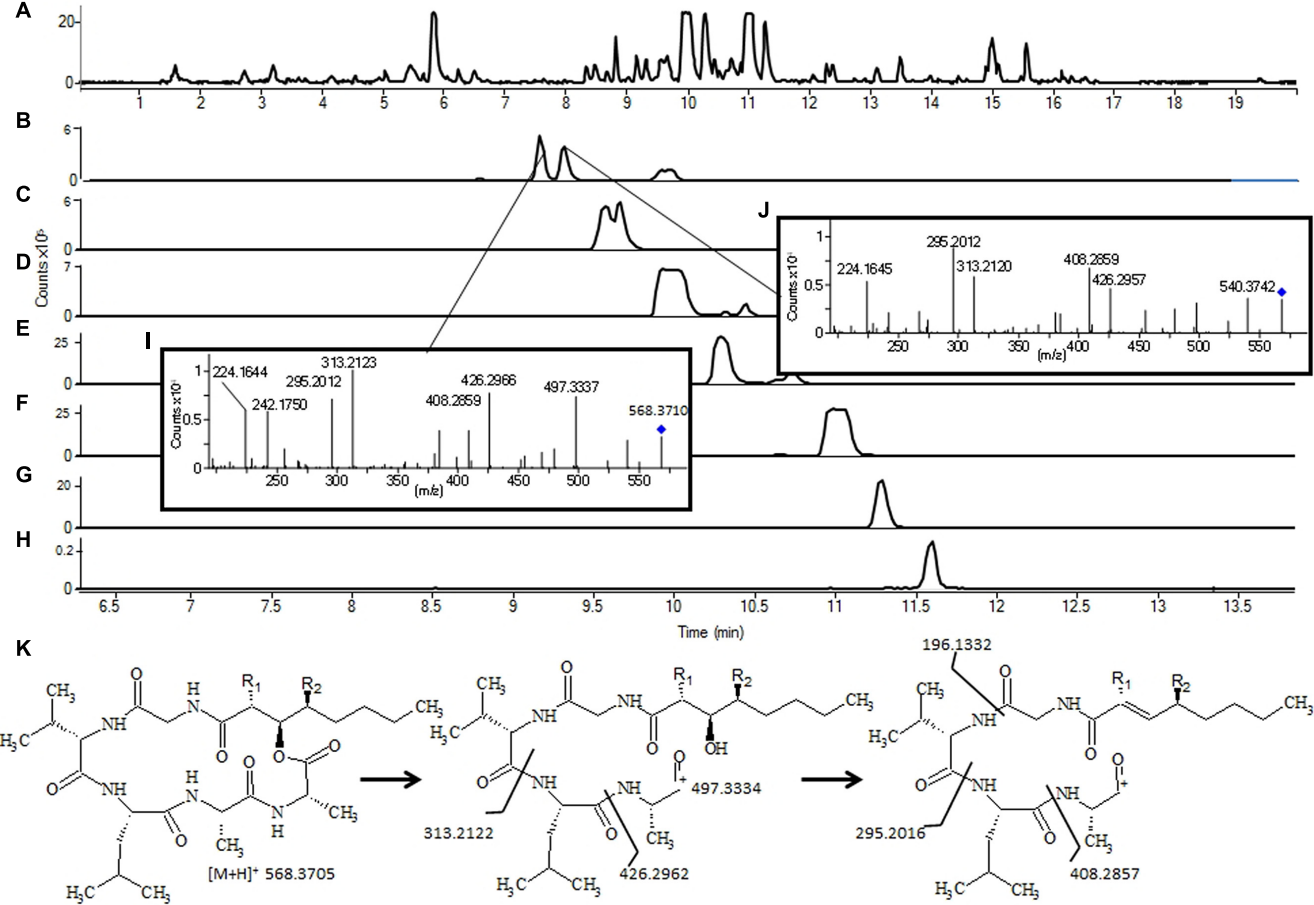
**Tentative identification of emericellamides in an extract of *Aspergillus nidulans* grown for 7 days on yeast extract sucrose agar: (**A)** Base Peak chromatogram; (**B)** novel emericellamides where MS/HRMS and retention time suggest the structure shown in **(K)** with **(I)** RT 9.17 (*R*_**1**_ = CH_**3**_, *R*_**2**_ = H) and **(J)** RT 9.33 (*R*_**1**_ = H, *R*_**2**_ = CH_**3**_).** Water loss from the fatty acid chain is more favored when *R*_1_ = H as a conjugation with the carbon can occur. **(C)** shows other novel emericellamides G (*R*_1_ = H, *R*_2_ = H) presumably with the fatty chain 2 × CH_2_ longer. **(H)**, emericellamide B; **(G)**, emericellamide H (emericellamide E, with *R*_1_ = CH_3_, *R*_2_ = CH_3_); **(F)**, emericellamide F; **(E)**, emericellamide A; and **(D)**, emericellamide C. For instrument settings see Kildgaard et al. (2014).

Manual interpretation of MS/HRMS spectra might even be used to predict the structure of unknown compounds based on careful inspection of the obtained fragmentation patterns. This approach is especially applicable for NRPs due to their sequential composition of amino acids (**Figure 6**).

For ion-traps users (especially IT-Orbi trap) the so called MS-trees offers an additional dimension to identification of a substructure of an analyte molecule by MS/MS/MS (MS^3^; Ridder et al., 2012; Rojas-Cherto et al., 2012), as fragment ions from the first MS/MS event subsequently can be further fragmented and matched in a MS/MS library for assigning parts of a given molecular structure.

## MS NETWORKING

In recent years the Dorrestein and Bandeira labs (Guthals et al., 2012; Watrous et al., 2012) have been key drivers toward development of new networking MS/MS approaches. Here MS/MS spectra of compounds in a given sample are compared pairwise and structurally related compounds are clustered based on the presence of similar fragments and neutral loses. In this way several compounds belonging to the same pathways have very convincingly been linked together including compounds which belong to both known and novel biosynthetic pathways (Watrous et al., 2012; Yang et al., 2013). A major current drawback is the lack of back-integration of raw data for evaluation of the corresponding full scan data and retention time, making detailed analysis extremely time consuming. Also source code is not available in an open-source format as, e.g., XC-MS and it is thus impossible to troubleshoot data in cases where the analysis fails.

## ISOTOPE LABELING FOR STRUCTURE AND BIOSYNTHESIS INVESTIGATION

The availability of LC-MS instruments and increased availability of stable isotope (^13^C, ^2^H, ^15^N, ^34^S) labeled substrates has made more reliable determination of the elemental composition of a given compound more robust through determination of the number of carbon (Bueschl et al., 2012), nitrogen (Bode et al., 2012) or sulfur atoms in the molecule (Brock et al., 2014). However, the technology is also promising for incorporation of precursors involved in PK and NRP biosynthetic studies (McIntyre et al., 1982, 1989; Townsend and Christensen, 1983; Steyn et al., 1984; Simpson, 1998; Baran et al., 2010; Bode et al., 2012; Fuchs et al., 2012; Proschak et al., 2013; Brock et al., 2014; Huang et al., 2014), as recently exploited for proving that yanuthone production in *A. niger* is produced via 6-methylsalisylic acid and not shikimic acid (Holm et al., 2014; Petersen et al., 2015).

## SURFACE TECHNIQUES

During the last 7 years surface desorption techniques have been introduced for secondary metabolite screening. These techniques can both ionize and detect surface excreted compounds and compounds in agar, for example, the identification of compounds present between colonies of different organisms for studying interactions. Current methods can be separated into two different sub families: desorption electrospray ionization mass spectrometry (DESI), direct analysis in real time (DART) and variants of them, and they are all soft ionization techniques (Watrous et al., 2012; Hsu et al., 2013; Bouslimani et al., 2014). DESI (Esquenazi et al., 2009) is based on charged droplets of organic solvent and a gas jet hitting the surface and then sampling ions close by (Gurdak et al., 2014), whereas DART uses a gas ionized by a plasma. Both have a poor spatial resolution (mm range) and can only be applied for major structures as DESI imprint of exudate droplets (Figueroa et al., 2014). Another major field of surface desorption techniques are the matrix assisted laser desorption techniques (MALDI). MALDI has a much higher spatial resolution (down to several 100 μm), but still not low enough to provide cellular resolution of fungi (Watrous and Dorrestein, 2011; Watrous et al., 2012; Bouslimani et al., 2014).

## CONCLUSION AND PERSPECTIVES

The recent advancements in separation sciences, high resolution mass spectrometry, as well as data mining tools have dramatically improved our possibilities for dereplication of fungal natural products in complex mixtures. At the same time these advances call for more automated methods for analysis since the amounts of data that can be generated on a single instrument in a short time is truly immense. Among other techniques, dereplication based on auto-MS/MS has proven to be very robust and effective on a given instrument. However, the use of MS/MS settings between manufactures and scoring algorithms are calling for standardized methods for both generation and comparison of MS/MS patterns.

The ultimate solution for future dereplication would be the construction of an open natural product database, where all relevant chemical and taxonomic information have been merged together. In particular this should include chromatographic and spectroscopic data (HR MS/MS, UV/Vis, NMR) as well as valid taxonomic information about the source organism, in many cases maybe even including linking biosynthetic origin of a given natural product to the related gene cluster.

## Conflict of Interest Statement

The authors declare that the research was conducted in the absence of any commercial or financial relationships that could be construed as a potential conflict of interest.

## Footnotes

1 http://www.r-project.org/

2 http://pubs.rsc.org/marinlit/

## References

[B1] Abdel-Mawgoud A. M. Aboulwafa M. M. Hassouna N. A. H. (2008). Characterization of surfactin produced by *Bacillus subtilis* isolate BS5. *Appl. Biochem. Biotechnol.* 150 289–303 10.1007/s12010-008-8153-z 18437297

[B2] Ali H. Ries M. I. Lankhorst P. P. van der Hoeven R. A. M. Schouten O. L. Noga M. (2014). A Non-canonical NRPS ss Involved in the synthesis of fungisporin and related hydrophobic cyclic tetrapeptides in *Penicillium chrysogenum*. *PLoS ONE* 9:e98212 10.1371/journal.pone.0098212.g001 PMC4041764 24887561

[B3] Andersen B. Nielsen K. F. Jarvis B. B. (2002). Characterization of *Stachybotrys* from water-damaged buildings based on morphology, growth and metabolite production. *Mycologia* 94 392–403 10.2307/3761773 21156510

[B4] Apfelthaler E. Bicker W. Lammerhofer M. Sulyok M. Krska R. Lindner W. (2008). Retention pattern profiling of fungal metabolites on mixed-mode reversed-phase/weak anion exchange stationary phases in comparison to reversed-phase and weak anion exchange separation materials by liquid chromatography-electrospray ionisation-tandem mass spectrometry. *J. Chromatogr. A* 1191 171–181 10.1016/j.chroma.2007.12.067 18199445

[B5] Baran R. Bowen B. P. Bouskill N. J. Brodie E. L. Yannone S. M. Northen T. R. (2010). Metabolite identification in *Synechococcus* sp. *PCC* 7002 using untargeted stable isotope assisted metabolite profiling. *Anal. Chem.* 82 9034–9042 10.1021/ac1020112 20945921

[B6] Baumann C. Cintora M. A. Eichler M. Lifante E. Cooke M. Przyborowska A. (2000). A library of atmospheric pressure ionization daughter ion mass spectra based on wideband excitation in an ion trap mass spectrometer. *Rapid Commun. Mass Spectrom.* 14 349–356 10.1002/(SICI)1097-0231(20000315)14:5<349::AID-RCM873>3.0.CO;2-P 10700037

[B7] Bergmann S. Schumann J. Scherlach K. Lange C. Brakhage A. A. Hertweck C. (2007). Genomics-driven discovery of PKS-NRPS hybrid metabolites from *Aspergillus nidulans*. *Nat. Chem. Biol.* 3 213–217 10.1038/nchembio869 17369821

[B8] Bertrand S. Schumpp O. Bohni N. Bujard A. Azzollini A. Monod M. (2013). Detection of metabolite induction in fungal co-cultures on solid media by high-throughput differential ultra-high pressure liquid chromatography-time-of-flight mass spectrometry fingerprinting. *J. Chromatogr. A* 1292 219–228 10.1016/j.chroma.2013.01.098 23466199

[B9] Bills G. F. (1995). Analysis of microfungal diversity from a user’s perspective. *Can. J. Bot.* 73 s33–s41 10.1139/b95-222

[B10] Bitzer J. Kopcke B. Stadler M. Heilwig V. Ju Y. M. Seip S. (2007). Accelerated dereplication of natural products, supported by reference libraries. *Chimia (Aarau)* 61 332–338 10.2533/chimia.2007.332

[B11] Bladt T. T. Duerr C. Knudsen P. B. Kildgaard S. Frisvad J. C. Gotfredsen C. H. (2013). Bio-Activity and dereplication-based discovery of ophiobolins and other fungal secondary metabolites targeting leukemia cells. *Molecules* 18 14629–14650 10.3390/molecules181214629 24287995 PMC6290568

[B12] Blunt J. W. Copp B. R. Hu W. P. Munro M. H. G. Northcote P. T. Prinsep M. R. (2008). Marine natural products. *Nat. Prod. Rep.* 25 35–94 10.1039/b701534h 18250897

[B13] Bode H. B. Bethe B. Höfs R. Zeeck A. (2002). Big effects from small changes: possible ways to explore Nature’s chemical diversity. *ChemBioChem* 3 619–627 10.1002/1439-7633(20020703) 12324995

[B14] Bode H. B. Reimer D. Fuchs S. W. Kirchner F. Dauth C. Kegler C. (2012). Determination of the absolute configuration of peptide natural products by using stable isotope labeling and mass spectrometry. *Chem. A Eur. J.* 18 2342–2348 10.1002/chem.201103479 22266804

[B15] Bok J. W. Chiang Y. M. Szewczyk E. Reyes-Domingez Y. Davidson A. D. Sanchez J. F. (2009). Chromatin-level regulation of biosynthetic gene clusters. *Nat. Chem. Biol.* 5 462–464 10.1038/nchembio.177 19448638 PMC2891026

[B16] Boonzaaijer G. Bobeldijk I. van Osenbruggen W. A. (2005). Analysis of patulin in dutch food, an evaluation of a SPE based method. *Food Control* 16 587–591 10.1016/j.foodcont.2004.06.020

[B17] Bouslimani A. Sanchez L. M. Garg N. Dorrestein P. C. (2014). Mass spectrometry of natural products: current, emerging, and future technologies. *Nat. Prod. Rep.* 31 718–729 10.1039/c4np00044g 24801551 PMC4161218

[B18] Brock N. L. Nikolay A. Dickschat J. S. (2014). Biosynthesis of the antibiotic tropodithietic acid by the marine bacterium *Phaeobacter inhibens*. *Chem. Commun.* 50 5487–5489 10.1039/c4cc01924e 24723119

[B19] Broecker S. Herre S. Wust B. Zweigenbaum J. Pragst F. (2011). Development and practical application of a library of CID accurate mass spectra of more than 2,500 toxic compounds for systematic toxicological analysis by LC-QTOF-MS with data-dependent acquisition. *Anal. Bioanal. Chem.* 400 101–117 10.1007/s00216-010-4450-9 21127842

[B20] Bueschl C. Kluger B. Berthiller F. Lirk G. Winkler S. Krska R. (2012). METEXTRACT: a new software tool for the automated comprehensive extraction of metabolite-derived LC/MS signals in metabolomics research. *Bioinformatics* 28 1–5 10.1093/bioinformatics/bts012 22238263 PMC3289915

[B21] Butler M. S. (2004). The role of natural product chemistry in drug discovery. *J. Nat. Prod.* 67 2141–2153 10.1021/np040106y 15620274

[B22] Cannall R. J. P. (1998). “Methods in biotechnolog,” in *Natural Products Isolation* Vol. 4 ed. Cannall R. J. P. (New Jersey: Humana Press) 1–51 10.1007/978-1-59259-256-2_1

[B23] Chen X. Cao Y. Lv D. Zhu Z. Zhang J. Chai Y. (2012). Comprehensive two-dimensional HepG2/cell membrane chromatography/monolithic column/time-of-flight mass spectrometry system for screening anti-tumor components from herbal medicines. *J. Chromatogr. A* 1242 67–74 10.1016/j.chroma.2012.04.034 22552199

[B24] Chiang Y. M. Szewczyk E. Davidson A. D. Entwistle R. Keller N. P. Wang C. C. C. (2010). Characterization of the *Aspergillus nidulans* monodictyphenone gene cluster. *Appl. Environ. Microbiol.* 76 2067–2074 10.1128/AEM.02187 20139316 PMC2849234

[B25] Cordell G. A. Shin Y. G. (1999). Finding the needle in the haystack. *The* dereplication of natural products extracts. *Pure Appl. Chem.* 71 1089–1094 10.1351/pac199971061089

[B26] Cox D. G. Oh J. Keasling A. Colson K. L. Hamann M. T. (2014). The utility of metabolomics in natural product and biomarker characterization. *Biochim. Biophys. Acta* 1840 3460–3474 10.1016/j.bbagen.2014.08.007 25151044 PMC4475408

[B27] Creek D. J. Jankevics A. Breitling R. Watson D. G. Barrett M. P. Burgess K. E. V. (2011). Toward global metabolomics analysis with hydrophilic interaction liquid chromatography-mass spectrometry: improved metabolite identification by retention time prediction. *Anal. Chem.* 83 8703–8710 10.1021/ac2021823 21928819

[B28] Degenkolb T. Grafenhan T. Nirenberg H. I. Gams W. Bruckner H. (2006). *Trichoderma* brevicompactum complex: rich source of novel and recurrent plant-protective polypeptide antibiotics (peptaibiotics). *J. Agric. Food Chem.* 54 7047–7061 10.1021/jf060788q 16968062

[B29] de la Cruz M. Martin J. Gonzalez-Menendez V. Perez-Victoria I. Moreno C. Tormo J. R. (2012). Chemical and physical modulation of antibiotic activity in *Emericella Species*. *Chem. Biodivers* 9 1095–1113 10.1002/cbdv.201100362 22700228

[B30] Dinan L. (2005). “Methods in biotechnology,” in *Dereplication and Partial Identification of Compounds Natural Products Isolation* 2nd Edn Vol. 20 eds Sarker S. D. Latif Z. Gray A. I. (Louisville, KY: Humana Press) 297–321.

[B31] Droce A. Sorensen J. L. Giese H. Sondergaard T. E. (2013). Glass bead cultivation of fungi: combining the best of liquid and agar media. *J. Microbiol. Methods.* 94 343–346 10.1016/j.mimet.2013.07.005 23871859

[B32] Dufresne C. (1998). “Methods in biotechnolog,” in *Isolation by Ion-Exchange Methods, Natural Products Isolation* Vol. 4 ed. Cannall R. J. P. (New Jersey: Humana Press) 141–164.

[B33] Dugo P. Cacciola F. Donato P. Airado-Rodriguez D. Herrero M. Mondello L. (2009). Comprehensive two-dimensional liquid chromatography to quantify polyphenols in red wines. *J. Chromatogr. A* 1216 7483–7487 10.1016/j.chroma.2009.04.001 19398102

[B34] El-Elimat T. Figueroa M. Ehrmann B. M. Cech N. B. Pearce C. J. Oberliesm N. H. (2013). High-resolution MS, MS/MS, and UV database of fungal secondary metabolites as a dereplication protocol for bioactive natural products. *J. Nat. Prod.* 76 1709–1716 10.1021/np4004307 23947912 PMC3856222

[B35] Esquenazi E. Dorrestein P. C. Gerwick W. H. (2009). Probing marine natural product defenses with DESI-imaging mass spectrometry. *Proc. Natl. Acad. Sci. U.S.A.* 106 7269–7270 10.1073/pnas.0902840106 19416917 PMC2678597

[B36] Euerby M. R. Petersson P. (2005). Chromatographic classification and comparison of commercially available reversed-phase liquid chromatographic columns containing polar embedded groups/amino endcappings using principal component analysis. *J. Chromatogr. A* 1088 1–15 10.1016/j.chroma.2004.10.027 16130727

[B37] Feng X. Siegel M. M. (2007). FTICR-MS applications for the structure determination of natural products. *Anal. Bioanal. Chem.* 389 1341–1363 10.1007/s00216-007-1468-8 17701030

[B38] Figueroa M. Jarmusch A. K. Raja H. A. El-Elimat T. Kavanaugh J. S. Horswill A. R. (2014). Polyhydroxyanthraquinones as quorum sensing inhibitors from the guttates of *Penicillium restrictum* and their analysis by desorption electrospray ionization mass spectrometry. *J. Nat. Prod.* 77 1351–1358 10.1021/np5000704 24911880 PMC4073659

[B39] Firn R. D. Jones C. G. (2003). Natural products - a simple model to explain chemical diversity. *Nat. Prod. Rep.* 20 382–391 10.1039/b208815k 12964834

[B40] Fredenhagen A. Derrien C. Gassmann E. (2005). An MS/MS library on an ion-trap instrument for efficient dereplication of natural products. Different fragmentation patterns for [M + H]+ and [M +Na]+ ions. *J. Nat. Prod.* 68 385–391 10.1021/np049657e 15787441

[B41] Fuchs S. W. Sachs C. C. Kegler C. Nollmann F. I. Karas M. Bode H. B. (2012). Neutral loss fragmentation pattern based screening for arginine-rich natural products in *Xenorhabdus* and *Photorhabdus*. *Anal. Chem.* 84 6948–6955 10.1021/ac300372p 22873683

[B42] Gurdak E. Green F. M. Rakowska P. D. Seah M. P. Salter T. L. R. Gilmore I. S. (2014). VAMAS interlaboratory study for desorption electrospray ionisa-tion mass spectrometry (DESI MS) intensity repeatability and constancy. *Anal. Chem.* 86 9603–9611 10.1021/ac502075t 25208328

[B43] Guthals A. Watrous J. D. Dorrestein P. C. Bandeira N. (2012). The spectral networks paradigm in high throughput mass spectrometry. *Mol. Biosyst.* 8 2535–2544 10.1039/c2mb25085c 22610447 PMC3893064

[B44] Hansen M. E. Smedsgaard J. Larsen T. O. (2005). X-Hitting: an algorithm for novelty detection and dereplication by UV spectra of complex mixtures of natural products. *Anal. Chem.* 77 6805–6817 10.1021/ac040191e 16255577

[B45] Hill A. W. Mortishire-Smith R. J. (2005). Automated assignment of high-resolution collisionally activated dissociation mass spectra using a systematic bond disconnection approach. *Rapid Commun. Mass Spectrom.* 19 3111–3118 10.1002/rcm.2177

[B46] Hinkley S. F. Jarvis B. B. (2000). “Methods molecular biology, in *Chromatographic Method for Stachybotrys Toxins,* *157. Mycotoxin Protocols* eds Pohland A. Trucksess M. W. (Totowa: Humana Press) 173–194 10.1385/1-59259-064-0:173 11051002

[B47] Holm D. K. Petersen L. M. Klitgaard A. Knudsen P. B. Jarczynska Z. Nielsen K. F. (2014). Molecular and chemical characterization of the biosynthesis of the 6-MSA-derived meroterpenoid yanuthone D in *Aspergillus niger*. *Chem. Biol.* 21 519–529 10.1016/j.chembiol.2014.01.013 24684908

[B48] Horai H. Arita M. Kanaya S. Nihei Y. Ikeda T. Suwa K. (2010). MassBank: a public repository for sharing mass spectral data for life sciences. *J. Mass Spectrom.* 45 703–714 10.1002/jms.1777 20623627

[B49] Hsu C. C. ElNaggar M. S. Peng Y. Fang J. Sanchez L. M. Mascuch S. J. (2013). Real-time metabolomics on living microorganisms using ambient electrospray ionization flow-probe. *Anal. Chem.* 85 7014–7018 10.1021/ac401613x 23819546 PMC3890442

[B50] Huang Y. Q. Wang Q. Y. Liu J. Q. Hao Y. H. Yuan B. F. Feng Y. Q. (2014). Isotope labelling - paired homologous double neutral loss scan-mass spectrometry for profiling of metabolites with a carboxyl group. *Analyst* 139 3446–3454 10.1039/c4an00312h 24839964

[B51] Hufsky F. Scheubert K. Bocker S. (2014). Computational mass spectrometry for small-molecule fragmentation. *Track-Trends Anal. Chem.* 53 41–48 10.1016/j.trac.2013.09.008

[B52] Kangas L. J. Metz T. O. Isaac G. Schrom B. T. Ginovska-Pangovska B. Wang L. N. (2012). In silico identification software (ISIS): a machine learning approach to tandem mass spectral identification of lipids. *Bioinformatics* 28 1705–1713 10.1093/bioinformatics/bts194 22592377 PMC3381961

[B53] Kildgaard S. Mansson M. Dosen I. Klitgaard A. Frisvad J. C. Larsen T. O. (2014). Accurate dereplication of bioactive secondary metabolites from marine-derived fungi by UHPLC-DAD-QTOFMS and a MS/HRMS library. *Mar. Drugs* 12 3681–3705 10.3390/md12063681 24955556 PMC4071597

[B54] Kind T. Fiehn O. (2007). Seven golden rules for heuristic filtering of molecular formulas obtained by accurate mass spectrometry. *BMC Bioinform.* 8:105 10.1186/1471-2105-8-105 PMC1851972 17389044

[B55] Klitgaard A. Iversen A. Andersen M. R. Larsen T. O. Frisvad J. C. Nielsen K. F. (2014). Aggressive dereplication using UHPLC-DAD-QTOF - screening extracts for up to 3000 fungal secondary metabolites. *Anal. Bioanal. Chem.* 406 1933–1943 10.1007/s00216-013-7582-x 24442010 PMC3955480

[B56] Kuhl C. Tautenhahn R. Boettcher C. Larson T. R. Neumann S. (2012). CAMERA: an integrated strategy for compound spectra extraction and annotation of liquid chromatography/mass spectrometry data sets. *Anal. Chem.* 84 283–289 10.1021/ac202450g 22111785 PMC3658281

[B57] Kvitvang H. F. N. Kristiansen K. A. Bruheim P. (2014). Assessment of capillary anion exchange ion chromatography tandem mass spectrometry for the quantitative profiling of the phosphometabolome and organic acids in biological extracts. *J. Chromatogr. A* 1370 70–79 10.1016/j.chroma.2014.10.029 25454131

[B58] Lammerhofer M. Richter M. Wu J. Y. Nogueira R. Bicker W. Lindner W. (2008). Mixed-mode ion-exchangers and their comparative chromatographic characterization in reversed-phase and hydrophilic interaction chromatography elution modes. *J. Sep. Sci.* 31 2572–2588 10.1002/jssc.200800178 18693304

[B59] Lang G. Mayhudin N. A. Mitova M. I. Sun L. van der Sar S. Blunt J. W. (2008). Evolving trends in the dereplication of natural product extracts: new methodology for rapid, small-scale investigation of natural product extracts. *J. Nat. Prod.* 71 1595–1599 10.1021/np8002222 18710284

[B60] Langseth W. Rundberget T. (1998). Instrumental methods for determination of nonmacrocyclic trichothecenes in cereals, foodstuffs and cultures. *J. Chromatogr. A* 815 103–121 10.1016/S0021-9673(98)00388-4

[B61] Larsen T. O. Franzyk H. Jensen S. R. (1999). UV-guiden isolation of verrucines A and B, novel quinazolines from *Penicillium verrucosum* structuraly related to anacine from *Penicillium aurantiogriseum*. *J. Nat. Prod.* 62 1578–1580 10.1021/np990251p 10579880

[B62] Larsen T. O. Petersen B. O. Duus J. O. Sørensen D. Frisvad J. C. Hansen M. E. (2005). Discovery of new natural products by application of X-hitting, a novel algorithm for automated comparison of full UV spectra, combined with structural determination by NMR spectroscopy. *J. Nat. Prod.* 68 871–874 10.1021/np040248s 15974610

[B63] Larsen T. O. Smedsgaard J. Nielsen K. F. Hansen M. E. Samson R. A. Frisvad J. C. (2007). Production of mycotoxins by *Aspergillus lentulus* and other medically important and closely related species in section *Fumigati*. *Med. Mycol.* 45 225–232 10.1080/13693780601185939 17464844

[B64] Lebrun M. H. Gaudemer F. Boutar M. Nicolas L. Goudemer A. (1989). Ion-pair, anion-exchange and ligand-excahange high-performance liquid chromatography of tenuazonic acid and 3-acetyl 5-substituted pyrrolidiene-2,4-diones. *J. Chromatogr.* 464 307–322 10.1016/S0021-9673(00)94249-3 2722982

[B65] Lee S. J. Kim D. H. Liu K. H. Oh T. K. Lee C. H. (2005). Identification of flavonoids using liquid chromatography with electrospray ionization and ion trap tandem mass spectrometry with an MS/MS library. *Rapid Commun. Mass Spectrom.* 19 3539–3548 10.1002/rcm.2230 16261653

[B66] Lehner S. M. Neumann N. K. N. Sulyok M. Lemmens M. Krska R. Schuhmacher R. (2011). Evaluation of LC-high-resolution FT-Orbitrap MS for the quantification of selected mycotoxins and the simultaneous screening of fungal metabolites in food. *Food Addit. Contam.* 28 1457–1468 10.1080/19440049.2011.599340 21854354

[B67] Lysoe E. Harris L. Subramaniam R. Divon H. H. Riiser E. S. Llorens C. (2014). The genome of the generalist plant pathogen *Fusarium avenaceum* is enriched with genes involved in redox, signaling, and secondary metabolism. *PLoS ONE* 9:e112703 10.1371/journal.pone.0112703 PMC4237347 25409087

[B68] Macintyre L. Zhang T. Viegelmann C. Martinez I. J. Cheng C. Dowdells C. (2014). Metabolomic tools for secondary metabolite discovery from marine microbial symbionts. *Mar. Drugs* 12 3416–3448 10.3390/md12063416 24905482 PMC4071584

[B69] Magdenoska O. Martinussen J. Thykaer J. Nielsen K. F. (2013). Dispersive solid phase extraction combined with ion-pair ultra high-performance liquid chromatography tandem mass spectrometry for quantification of nucleotides in *Lactococcus lactis*. *Anal. Biochem.* 440 166–177 10.1016/j.ab.2013.05.023 23747533

[B70] Månsson M. Phipps R. K. Gram L. Munro M. H. Larsen T. O. Nielsen K. F. (2010). Explorative solid-phase extraction (E-SPE) for accelerated microbial natural product discovery, dereplication, and purification. *J. Nat. Prod.* 73 1126–1132 10.1021/np100151y 20509666

[B71] May J. C. Goodwin C. R. Lareau N. M. Leaptrot K. L. Morris C. B. Kurulugama R. T. (2014). Conformational ordering of biomolecules in the gas phase: nitrogen collision cross sections measured on a prototype high resolution drift tube ion mobility-mass spectrometer. *Anal. Chem.* 86 2107–2116 10.1021/ac4038448 24446877 PMC3931330

[B72] McIntyre C. R. Scott F. E. Simpson T. J. Trimble L. A. Vederas J. C. (1989). Application of stable isotope to labeling methodology the biosynthesis of the mycotoxin, terretonin, by *Aspergillus terreus* - incorporation of C-13-labeled acetates and methionine, H-2-labeled and C-13-labeled, O-18-labeled ethyl 3,5-dimethylorsellinate and O-18 gas. *Tetrahedron* 45 2307–2321 10.1016/S0040-4020(01)83433-5

[B73] McIntyre C. R. Simpson T. J. Stenzel D. J. Bartlett A. J. O’Brien E. O. (1982). Biosynthsis of the meroterpenoid metabolites. austin and terretonin: incoporation of 3,5-dimethylorsellinate. *J. Chem. Soc. Chem. Commun.* 781–782 10.1039/c39820000781

[B74] Miller J. D. (2008). Mycotoxins in small grains and maize: old problems, new challenges. *Food Addit. Contam.* 25 219–230 10.1080/02652030701744520 18286412

[B75] Mogensen J. M. Sørensen S. M. Sulyok M. van der Westhuizen L. Shepard G. S. Frisvad J. C. (2011). Single kernel analysis of fumonisins and other fungal metabolites in maize from South African subsistence farmers. *Food Addit. Contam.* 28 1724–1734 10.1080/19440049.2011.611823 22023397

[B76] Nagao T. Yukihira D. Fujimura Y. Saito K. Takahashi K. Miura D. (2014). Power of isotopic fine structure for unambiguous determination of metabolite elemental compositions: in silico evaluation and metabolomic application. *Anal. Chim. Acta* 813 70–76 10.1016/j.aca.2014.01.032 24528662

[B77] Nielsen K. F. Frisvad J. C. (2014). *DTU Mycotoxin-Fungal Secondary Metabolite MS/HRMS Library*. Available at: http://www.bio.dtu.dk/english/Research/Platforms/Metabolom/MSMSLib

[B78] Nielsen K. F. Månsson M. Rank C. Frisvad J. C. Larsen T. O. (2011a). Dereplication of microbial natural products by LC-DAD-TOFMS. *J. Nat. Prod.* 74 2338–2348 10.1021/np200254t 22026385

[B79] Nielsen M. L. Nielsen J. B. Rank C. Klejnstrup M. L. Holm D. K. Brogaard K. H. (2011b). A genome-wide polyketide synthase deletion library uncovers novel genetic links to polyketides and meroterpenoids in *Aspergillus nidulans*. *FEMS Microbiol. Lett.* 321 157–166 10.1111/j.1574-6968.2011.02327.x 21658102

[B80] Nielsen K. F. Mogensen J. M. Johansen M. Larsen T. O. Frisvad J. C. (2009). Review of secondary metabolites and mycotoxins from the *Aspergillus niger* group. *Anal. Bioanal. Chem.* 395 1225–1242 10.1007/s00216-009-3081-5 19756540

[B81] Nielsen K. F. Ngemela A. F. Jensen L. B. de Medeiros L. S. Rasmussen P. H. (2015). UHPLC-MS/MS determination of ochratoxin A and fumonisins in coffee in using QuEChERS extraction combined with mixed-mode SPE purification. *J. Agric. Food Chem.* 63 1029–1034 10.1021/jf504254q 25553918

[B82] Nielsen K. F. Smedsgaard J. (2003). Fungal metabolite screening: database of 474 mycotoxins and fungal metabolites for de-replication by standardised liquid chromatography-UV-mass spectrometry methodology. *J. Chromatogr. A* 1002 111–136 10.1016/S0021-9673(03)00490-4 12885084

[B83] Oberacher H. Pavlic M. Libiseller K. Schubert B. Sulyok M. Schuhmacher R. (2009). On the inter-instrument and inter-laboratory transferability of a tandem mass spectral reference library: 1. Results of an Austrian multicenter study. *J. Mass Spectrom.* 44 485–493 10.1002/jms.1545 19165818

[B84] Oberacher H. Pitterl F. Siapi E. Steele B. R. Letzel T. Grosse S. (2012). On the inter-instrument and the inter-laboratory transferability of a tandem mass spectral reference library. 3. Focus on ion trap and upfront CID. *J. Mass Spectrom.* 47 263–270 10.1002/jms.2961 22359338

[B85] Oberacher H. Weinmann W. Dresen S. (2011). Quality evaluation of tandem mass spectral libraries. *Anal. Bioanal. Chem.* 400 2641–2648 10.1007/s00216-010-4598-3 21369757

[B86] Ojanpera S. Pelander A. Pelzing M. Krebs I. Vuori E. Ojanpera I. (2006). Isotopic pattern and accurate mass determination in urine drug screening by liquid chromatography/time-of-flight mass spectrometry. *Rapid Commun. Mass Spectrom.* 20 1161–1167 10.1002/rcm.2429 16521169

[B87] Pavlic M. Libiseller K. Oberacher H. (2006). Combined use of Esi-Qqtof-Ms and Esi-Qqtof-Ms/Ms with mass-spectral library search for qualitative analysis of drugs. *Anal. Bioanal. Chem.* 386 69–82 10.1007/s00216-006-0634-8 16896628

[B88] Perry R. H. Cooks R. Noll R. J. (2008). Orbitrap mass spectrometry: instrumentation, ion motion, and applications. *Mass Spectrom. Rev.* 27 661–699 10.1002/mas.20186 18683895

[B89] Petersen L. M. Holm D. K. Knudsen P. B. Nielsen K. F. Godtfredsen C. H. Mortensen U. H. (2015). Characterization of four novel antifungal yanuthones from *Aspergillus niger*. *J. Antibiot. On Line* 10.1038/ja.2014.130 [Epub ahead of print]. 25293978

[B90] Proschak A. Lubuta P. Grun P. Lohr F. Wilharm G. De Berardinis V. (2013). Structure and biosynthesis of fimsbactins A-F, siderophores from *Acinetobacter baumannii* and *Acinetobacter baylyi*. *ChemBioChem* 14 633–638 10.1002/cbic.201200764 23456955

[B91] Pucci V. Di Palma S. Alfieri A. Bonelli F. Monteagudo E. (2009). A novel strategy for reducing phospholipids-based matrix effect in LC-ESI-MS bioanalysis by means of HybridSPE. *J. Pharmaceut. Biomed. Anal.* 50 867–871 10.1016/j.jpba.2009.05.037 19553055

[B92] Rasmussen B. B. Nielsen K. F. Machado H. Gram L. Sonnenschein E. (2014). Global and phylogenetic distribution of quorum sensing signals, acyl homoserine lactones, in the family of Vibrionaceae. *Mar. Drugs* 12 5527–5546 10.3390/md12115527 25419995 PMC4245543

[B93] Ridder L. van der Hooft J. J. J. Verhoeven S. De Vos R. C. H. van Schaik R. Vervoort J. (2012). Substructure-based annotation of high-resolution multistage MSn spectral trees. *Rapid Commun. Mass Spectrom.* 26 2461–2471 10.1002/rcm.6364 22976213

[B94] Rojas-Cherto M. Peironcely J. E. Kasper P. T. van der Hooft J. J. J. De Vos R. C. H. Vreeken R. (2012). Metabolite identification using automated comparison of high-resolution multistage mass spectral trees. *Anal. Chem.* 84 5524–5534 10.1021/ac2034216 22612383

[B95] Rundberget T. Skaar I. O’Brien O. Flåøyen A. (2004). Penitrem and thomitrem formation by *Penicillium crustosum*. *Mycopathologia* 157 349–357 10.1023/B:MYCO.0000024180.99262.b1 15180164

[B96] Sanchez J. F. Somoza A. D. Keller N. P. Wang C. C. C. (2012). Advances in *Aspergillus* secondary metabolite research in the post-genomic era. *Nat. Prod. Rep.* 29 351–371 10.1039/c2np00084a 22228366 PMC4568942

[B97] Sarkara A. Funka A. N. Scherlach K. Horn F. Schroeckh V. Chankhamjon P. (2012). Differential expression of silent polyketide biosynthesis gene clusters in chemostat cultures of *Aspergillus nidulans*. *J. Biotechnol.* 160 64–71 10.1016/j.jbiotec.2012.01.015 22306112

[B98] Schroeckh V. Scherlach K. Nützmann H.-W. Shelest E. Schmidt-Heck W. Schuemann J. (2009). Intimate bacterial–fungal interaction triggers biosynthesis of archetypal polyketides in *Aspergillus nidulans*. *Proc. Natl. Acad. Sci. U.S.A* 106 14558–14563 10.1073/pnas.0901870106 19666480 PMC2732885

[B99] Shephard G. S. (2008). Impact of mycotoxins on human health in developing countries. *Food Addit. Contam.* 25 146–151 10.1080/02652030701567442 18286404

[B100] Shimizu Y. (1998). “Methods in biotechnolog,” in *Natural Products Isolation, Purification of Water-Soluble Products* Vol. 4 ed. Cannall R. J. P. (New Jersey: Humana Press) 329–341.

[B101] Simpson T. J. (1998). *Application of Isotopic Methods to Secondary Metabolic Pathways Topics in Current Chemistry* Vol. 195 (Heidelberg: Springer) 1–48.

[B102] Sleno L. (2012). The use of mass defect in modern mass spectrometry. *J. Mass Spectrom.* 47 226–236 10.1002/jms.2953 22359333

[B103] Smith C. A. O’Maille G. Want E. J. Qin C. Trauger S. A. Brandon T. R. (2005). METLIN: a metabolite mass spectral database. *Ther. Drug Monit.* 27 747–751 10.1097/01.ftd.0000179845.53213.39 16404815

[B104] Smith C. A. Want E. J. O’Maille G. Abagyan R. Siuzdak G. (2006). XCMS: Processing mass spectrometry data for metabolite profiling using Nonlinear peak alignment, matching, and identification. *Anal. Chem.* 78 779–787 10.1021/ac051437y 16448051

[B105] Sørensen J. L. Hansen F. T. Sondergaard T. E. Staerk D. Lee T. V. Wimmer R. (2012). Production of novel fusarielins by ectopic activation of the polyketide synthase 9 cluster in *Fusarium graminearum*. *Environ. Microbiol.* 14 1159–11570 10.1111/j.1462-2920.2011.02696.x 22252016

[B106] Sørensen J. L. Nielsen K. F. Thrane U. (2007). Analysis of moniliformin in maize plants using hydrophilic interaction chromatography. *J. Agric. Food Chem.* 55 9764–9768 10.1021/jf0715875 17960879

[B107] Sørensen J. L. Phipps R. K. Nielsen K. F. Schroers H. J. Frank J. Thrane U. (2009). Analysis of *Fusarium avenaceum* metabolites produced during wet apple core rot. *J. Agric. Food Chem.* 57 1632–1639 10.1021/jf802926u 19170495

[B108] Sorensen J. L. Sondergaard T. E. (2014). The effects of different yeast extracts on secondary metabolite production in *Fusarium*. *Int. J. Food Microbiol.* 170 55–60 10.1016/j.ijfoodmicro.2013.10.024 24291181

[B109] Stadler M. Fournier J. Quang D. N. Akulov A. Y. (2007). Metabolomic studies on the chemical ecology of the Xylariaceae (Ascomycota). *Nat. Prod. Commun.* 2 287–304.

[B110] Stadler M. Laessoe T. Fournier J. Decock C. Schmieschek B. Tichy H. V. (2014). A polyphasic taxonomy of *Daldinia* (Xylariaceae). *Stud. Mycol.* 77 1–143 10.3114/sim0016 24790283 PMC3953824

[B111] Stadler M. Ticky H. V. Katsiou E. Hellwig V. (2009). Chemotaxonomy of *Pochonia* and other conidial fungi with Verticillium-like anamorphs. *Mycol. Progress* 2 95–122 10.1007/s11557-006-0048-1

[B112] Steyn P. S. Vleggaar R. Simpson T. J. (1984). Stable isotope labelling studies on the biosynthesis of asticolorin-C by *Aspergillus multicolor* - evidence for a symmetrical intermediate. *J. Chem. Soc. Chem. Commun.* 765–767 10.1039/c39840000765

[B113] Sulyok M. Berthiller F. Krska R. Schuhmacher R. (2006). Development and validation of a liquid chromatography/tandem mass spectrometric method for the determination of 39 mycotoxins in wheat and maize. *Rapid Commun. Mass Spectrom.* 20 2649–2659 10.1002/rcm.2640 16912987

[B114] Townsend C. A. Christensen S. B. (1983). Stable isotope studies of anthraquinone intermediates in the aflatoxin pathway. *Tetrahedron* 39 3575–3582 10.1016/S0040-4020(01)88668-3

[B115] Tsunematsu Y. Ishikawa N. Wakana D. Goda Y. Noguchi H. Moriya H. (2013). Distinct mechanisms for spiro-carbon formation reveal biosynthetic pathway crosstalk. *Nat. Chem. Biol.* 9 818–827 10.1038/nchembio.1366 24121553

[B116] van Dam J. C. Eman M. R. Frank J. Lange H. C. van Dedem G. W. K. Heijnen S. J. (2002). Analysis of glycolytic intermediates in *Saccharomyces cerevisiae* using anion exchange chromatography and electrospray ionization with tandem mass spectrometric detection. *Anal. Chim. Acta* 460 209–218 10.1016/S0003-2670(02)00240-4

[B117] Varga E. Glauner T. Berthiller F. Krska R. Schuhmacher R. Sulyok M. (2013). Development and validation of a (semi-)quantitative UHPLC-MS/MS method for the determination of 191 mycotoxins and other fungal metabolites in almonds, hazelnuts, peanuts, and pistachios. *Anal. Bioanal. Chem.* 405 5087–5104 10.1007/s00216-013-6831-3 23471368 PMC3656230

[B118] Watrous J. D. Dorrestein P. C. (2011). Imaging mass spectrometry in microbiology. *Nat. Rev. Microbiol.* 9 683–694 10.1038/nrmicro2634 21822293 PMC3710447

[B119] Watrous J. Roach P. Alexandrov T. Heath B. S. Yang J. Y. Kersten R. D. (2012). Mass spectral molecular networking of living microbial colonies. *Proc. Natl. Acad. Sci. U.S.A.* 109 E1743–E1752 10.1073/pnas.1203689109 22586093 PMC3387089

[B120] Wehrens R. Carvalho E. Fraser P. (2014). Metabolite profiling in LC-DAD using multivariate curve resolution: the alsace package for R. *Metabolomics* 11 143–154 10.1007/s11306-014-0683-5

[B121] Werner A. (1993). Reversed-phase and ion-pair separations of nucleotides, nucleosides, and nucleobases - analysis of biological samples in health and disease. *J. Chromatogr. Biomed. Appl.* 618 3–14 10.1016/0378-4347(93)80024-X 8227262

[B122] Wolfender J. L. Marti G. Thomas A. l. Bertrand S. (2015). Current approaches and challenges for the metabolite profiling of complex natural extracts. *J. Chromatogr. A On Line* 10.1016/j.chroma.2014.10.091 [Epub ahead of print]. 25464997

[B123] Wurtinger P. Oberacher H. (2012). Evaluation of the performance of a tandem mass spectral library with mass spectral data extracted from literature. *Drug Test. Anal.* 4 235–241 10.1002/dta.341 21964810

[B124] Xian F. Hendrickson C. L. Marshall A. G. (2012). High resolution mass spectrometry. *Anal. Chem.* 84 708–719 10.1021/ac203191t 22263633

[B125] Yang J. Liang Q. Wang M. Jeffries C. Smithson D. Tu Y. (2014). UPLC-MS-ELSD-PDA as a powerful dereplication tool to facilitate compound identification from small-molecule natural product libraries. *J. Nat. Prod.* 77 902–909 10.1021/np4009706 24617915 PMC4784093

[B126] Yang J. Y. Sanchez L. M. Rath C. M. Liu X. Boudreau P. D. Bruns N. (2013). Molecular networking as a dereplication strategy. *J. Nat. Prod.* 76 1686–1699 10.1021/np400413s 24025162 PMC3936340

[B127] Yokota K. Yatsuda M. Miwa E. Higuchi K. (2012). Comparative study on sample preparation methods for the HPLC quantification of iturin from culture supernatant of an antagonistic *Bacillus* strain. *J. ISSAAS* 18 70–75.

[B128] Zengler K. Paradkar A. Keller M. (2009). “Natural products: drug discovery and therapeutic medicine, in *New Methods to Access Microbial Diversity for Small Molecule Discovery* eds Zhang L. Demain A. L. (Totowa, NJ: Humana Press Inc.) 275–293.

[B129] Zhang L. (2005). “Natural products: drug discovery and therapeutic medicine, in *Integrated Approaches for Discovering Novel Drugs from Microbial Natural Products* eds Zhang L. Demain A. L. (Totowa, NJ: Humana Press Inc.) 33–55.

[B130] Zhang X. M. Clausen M. R. Zhao X. L. Zheng H. Bertram H. C. (2012). Enhancing the power of liquid chromatography-mass spectrometry-based urine metabolomics in negative ion mode by optimization of the additive. *Anal. Chem.* 84 7785–7792 10.1021/ac3013835 22888765

